# Low-complexity regions in fungi display functional groups and are depleted in positively charged amino acids

**DOI:** 10.1093/nargab/lqaf014

**Published:** 2025-02-27

**Authors:** Kamil Steczkiewicz, Aleksander Kossakowski, Stanisław Janik, Anna Muszewska

**Affiliations:** Institute of Biochemistry and Biophysics, Polish Academy of Sciences, Pawinskiego 5A, 02-106 Warsaw, Poland; Institute of Biochemistry and Biophysics, Polish Academy of Sciences, Pawinskiego 5A, 02-106 Warsaw, Poland; Institute of Biochemistry and Biophysics, Polish Academy of Sciences, Pawinskiego 5A, 02-106 Warsaw, Poland; Faculty of Mathematics, Informatics and Mechanics, University of Warsaw, Stefana Banacha 2, 02-097 Warsaw, Poland; Institute of Biochemistry and Biophysics, Polish Academy of Sciences, Pawinskiego 5A, 02-106 Warsaw, Poland

## Abstract

Reports on the diversity and occurrence of low-complexity regions (LCR) in Eukaryota are limited. Some studies have provided a more extensive characterization of LCR proteins in prokaryotes. There is a growing body of knowledge about a plethora of biological functions attributable to LCRs. However, it is hard to determine to what extent observed phenomena apply to fungi since most studies of fungal LCRs were limited to model yeasts. To fill this gap, we performed a survey of LCRs in proteins across all fungal tree of life branches. We show that the abundance of LCRs and the abundance of proteins with LCRs are positively correlated with proteome size. We observed that most LCRs are present in proteins with protein domains but do not overlap with the domain regions. LCRs are associated with many duplicated protein domains. The quantity of particular amino acids in LCRs deviates from the background frequency with a clear over-representation of amino acids with functional groups and a negative charge. Moreover, we discovered that each lineage of fungi favors distinct LCRs expansions. Early diverging fungal lineages differ in LCR abundance and composition pointing at a different evolutionary trajectory of each fungal group.

## Introduction

Over three-quarters of protein sequences stored in Uniprot contain at least one protein domain (a structurally and functionally conserved region), summing up to 51% of total residue count [1]. The remaining proteins may either have very diverged, nondetectable domains, domains of yet unknown type, or are lacking protein domains at all. The research so far focused mainly on the domain-containing regions of proteins but more and more questions arise about the function of the remaining regions of protein sequences. In contrast to protein domains characterized with well defined amino acid compositions determining their 3D structure and function, the nondomain realm remains compositionally biased [2, 3] and requires an alternative approach to its functional annotation. The mainstream sequence analysis methods are designed to handle protein domains and firmly rely on propagating information patterns from either amino acid sequences alone or sequence profiles. In consequence, they remain ineffective when considering nondomain regions [4]. Despite the fundamental difficulties, compositionally biased sequences are being cataloged regarding their detailed characteristics. For instance, low-complexity regions (LCRs) consist of a limited number of residue species [2] and likely remain unstructured as intricate hydrophobic patterns are required to guide proper protein folding pathways. LCRs may however adopt local secondary structures [5].

Several attempts to study the amino acid composition of proteomes [6] or specific sequences, e.g. pentapeptides [7] showed a considerable number of compositionally biased regions with statistical properties differing from the background. Several general features of low-complexity regions have been recently systematically described by the community working on nonglobular proteins [2]. First, many of these regions overlap with single-residue tracts (homopolymers) or repeat regions, either simple or more complex. Second, some of them adopt coiled-coil structures or are intrinsically disordered [8]. Third, LCRs are often enriched in nonhydrophobic amino acid residues which predispose them to interactions and regulatory functions [5, 9].

The roles of LCRs in protein function, protein evolution or organism evolution remain understudied. However, we can draw some conclusions based on their unique characteristics. For instance, sequence repeats can expand due to polymerase slippage, which virtually can lead to protein coding sequence extension [10]. Long enough stretches of repeats can impact recombination and increase the mutation rate. Stretches of a single amino acid residue are widely present in viruses and eukaryotic organisms [6]. Repeats are particularly abundant in virulence factors of pathogenic agents, toxins, and allergens [11]. Tandem repeats are found in both noncoding and coding genomic regions and cause severe problems in assembly and annotation [12]. Many studies revealed the involvement of repeat regions in sophisticated regulatory mechanisms, and their tendency to expand over time [13].

Although generally unstructured, LCRs may form highly organized forms, like α-helical coiled-coils which are one of the most structurally varied forms of proteins [14]. Despite conceptual simplicity, they form rods, segmented ropes, barrels, funnels, sheets, spirals, and rings depending on the number of helices [8]. Proteins containing coiled-coil structures can function as molecular motors, are involved in DNA stabilization, and can transduce conformational changes [8]. In fungi, one of the core molecular clock proteins, *frq* (frequency clock protein), has a serine-rich coiled-coil region important for its functioning. The *fqr* protein is conserved beyond Dikarya and was found also in *Rhizophagus irregularis* (Glomeromycotina) [15]. Septins are yet another fungi-conserved class of glutamine-rich coiled-coil proteins which form oligomeric structures [16].

Intrinsically disordered proteins/regions (IDPs/IDRs) lack fixed 3D structure. IDRs are usually deficient in hydrophobic residues and are enriched in polar and charged residues [17, 18]. IDRs are essential for a wide range of biological functions, such as regulatory processes and cell signaling [18, 19]. In fungi, one of the prominent examples is *Saccharomyces cerevisiae* Knr4 protein involved in the crosstalk between cell wall integrity and calcineurin pathways [20]. Other IDPs may aggregate at septal pores controlling diverse aspects of intercellular connectivity [21]. Contrary to what’s generally observed for protein domains, sequence divergence of IDRs does not immediately mean different functions. On a proteome scale, there is evidence for shared functions between the highly diverged IDRs in *S. cerevisiae*, pointing at evolutionary selection acting differently on folded and disordered regions [17].

Current knowledge on LCRs functions comes from studying prokaryotic [9] and parasitic organisms [10]. However, eukaryotic proteomes not only contain LCRs but even seem to be enriched in these compared to prokaryotes [13]. Fungi, as model eukaryotes, are massively sequenced; therefore, we benefit from the abundance of complete genomes and predicted proteomes. Moreover, as a very diverse group of organisms, fungi are not only interesting for experts but also important for human health and the economy (with two faces as devastating pathogens of crop and beneficial fermenting organisms). The presence of particular protein domains may reflect the fungal lifestyle ability to interact with the environment [22]. On the other hand, the contribution of nondomain proteins to adaptability, ecology or rough functional categories remains elusive. LCRs are encountered all over the fungal protein universe and are associated with many expanded protein families. In the course of this study, we explored the distribution of LCRs within the fungal tree of life (FToL) to reconcile it with contemporary knowledge on fungal lineages.

## Materials and methods

The proteome and genomic sequences of 183 fungal species (*n* = 148 non-Dikarya and *n* = 35 Dikarya) were retrieved in May 2022 from the NCBI website [23] and assigned to phyla based on Spatafora *et al.* [24]. All assemblies were scanned with BUSCO v 5.7.1 against the fungi_odb10 set to assess their quality [25]. Microsporidia assemblies were additionally scanned against microsporidia_odb10 because of their low BUSCO scores when counting single copy orthologs from fungi_odb10. We verified that BUSCO scores were not correlated with LCR properties. Protein sets from assemblies with low BUSCO scores grouped together with sets of proteins from their closest relative with good BUSCO scores. In consequence, we decided to keep all the data. The BUSCO scores together with the N50 and L50 assembly parameters retrieved from NCBI are listed in Supplementary Table S1. Each fungal proteome was mapped on Pfam 36 [1] with pfamscan.pl with default setting to identify all protein domains in all of the proteins. All proteins with hits to Pfam were considered proteins with domains. Proteins with and without domains were analyzed and compared as two sets. Proteins with Pfam domains were also annotated with respective GO terms by PFAM2GO (version May 9, 2023) as reference [26]. All proteins with and without domains were scanned for the presence of sequence features such as transmembrane helices, signal peptides, and LCRs. Transmembrane helices were predicted with TMHMM2 v2.0c [27] and signal peptides with SignalP 5.0b [28]. SEG [29] software was used to find low-complexity regions. In order to reduce false positives a moderate set of parameters in SEG (1997 year edition) was used (*W* = 15, *K*1 = 1.9 and *K*2 = 2.5) following the experiences of the developers of PlaToLoCo web server (URL: https://platoloco.aei.polsl.pl/#!/help last accessed on January 24, 2025) [30] and published previously by Huntley and Golding [31]. LCRs were considered as homopolymers when they had at least five consecutive identical amino acids and this amino acid constituted at least 70% of the whole LCRs length. The frequencies of amino acids of LCRs were compared with the Uniprot statistics https://www.uniprot.org/uniprotkb/statistics (last accessed November 26, 2024) as well as with frequency in the set of all amino acid residues in the 183 proteomes, which we labeled fungal background amino acid frequencies.

The dataset consists of a TAB formatted table (Main Dataset.tsv) and derived spreadsheets (Supplementary Table S1). The Main Dataset integrates information about protein domains (pfam_scan, GO terms), LCRs (SEG), signal peptides, and transmembrane helices (TMHMM) for each of the analyzed proteins within 183 fungal proteomes. A perl script to integrate this information is available in Supplementary File S2. The Main Dataset has been designed to ease further searches with Linux bash commands, for e.g. sorting and subsetting by the aforementioned traits. Information on the taxonomical assignment and genomic publication were gathered manually from literature. The list of proteomes with taxonomical assignment and references to the genomic publications is listed in Supplementary Table S1. This resource can be used by users interested in detailed annotation of particular protein families, sets of organisms, LCRs, and types of proteins (for instance proteins with transmembrane helices).

LCRs were considered as overlapping a protein domain if 80% of LCRs amino acids were located within the domain region. Charts were drawn with the usage of the following Python’s [32] libraries: Pandas 2.2.2 [33], Matplotlib 3.8.4 [34], and Seaborn 0.13.2 [35]. A jupyter notebook listing the commands used to draw figures is available as Supplementary File S2 [36]. Chi-squared contingency tables were used for multiple comparisons and Pearson's correlation coefficient was used to assess the linear relationship between two datasets.

Multiple sequence alignments were calculated with MAFFT as a local alignment with an iterative refinement procedure (v7.407) [37] (localpair, maxitrate 100). Protein sequence alignments were visualized in seaview v. 4 [38] and were trimmed with TrimAl v1.4 with the gappyout option [39]. Maximum likelihood phylogenetic trees were constructed with IQ-TREE (v1.6.9, -m, -B 100) [40] with automated model selection and ultrafast bootstrap.

## Results

### LCRs occur commonly in proteins with distinguishable protein domains

**Figure 1.**
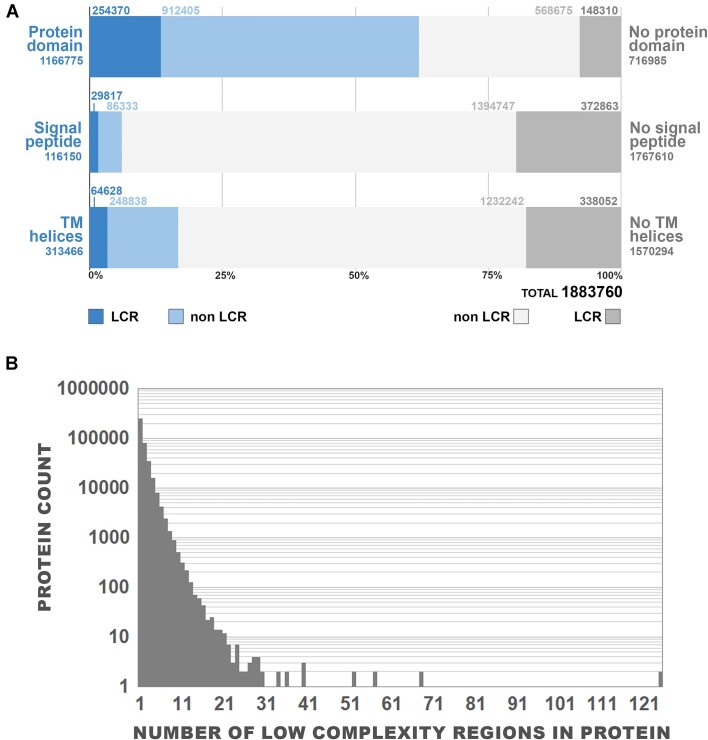
LCRs are present in fungal proteins with and without Pfam domain. (**A**) The counts of proteins containing LCRs together with either protein domains, signal peptides, or transmembrane regions. Numbers provided denote protein counts in each category. (**B**) Distribution of the number of LCR per protein in proteins having at least one LCR.

The first question which arises when considering LCRs is their overall abundance and co-occurrence with protein domains and other biologically relevant protein features, like signal peptides or transmembrane helices. LCRs make up 2.52% of the total length of proteins in analyzed fungal proteomes. Proteins with domains constitute 61.94% of all proteins (Fig. 1A), of which 21.8% (13.5% of all proteins) contain at least one LCR, comparable to 20.69% (7.87% of total count) for nondomain proteins. 6.17% of proteins have an N-terminal signal peptide, 25.67% (1.8% total) with LCR elements. 16.64% of all proteins contain at least one predicted transmembrane helix not overlapping with signal peptide. 20.62% (3.43% total) of membrane-tethered proteins have LCRs elements.

LCR abundance does not correlate with any other protein features such as presence of transmembrane helices, domains, or signal peptides (Supplementary Fig. S1). Most proteins have a single LCR (Fig. 1B); however, there are a few long proteins with over 10 LCR regions with the extreme case of KAG4098218.1 protein in anaerobic fungus *Neocallimastix* sp. JGI-2020a having 125 LCRs. This protein is 16 605 residues long and contains multiple repeats. Some proteins would report artifactual LCRs due to lengthy stretches of X’s marking unresolved regions in their sequences (for instance the truffle *Tuber melanosporum* protein CAZ85992.1), so we removed all sequences containing at least one tract of >5X symbols.

### Proteome size is a major determinant of the abundance of LCRs

**Figure 2.**
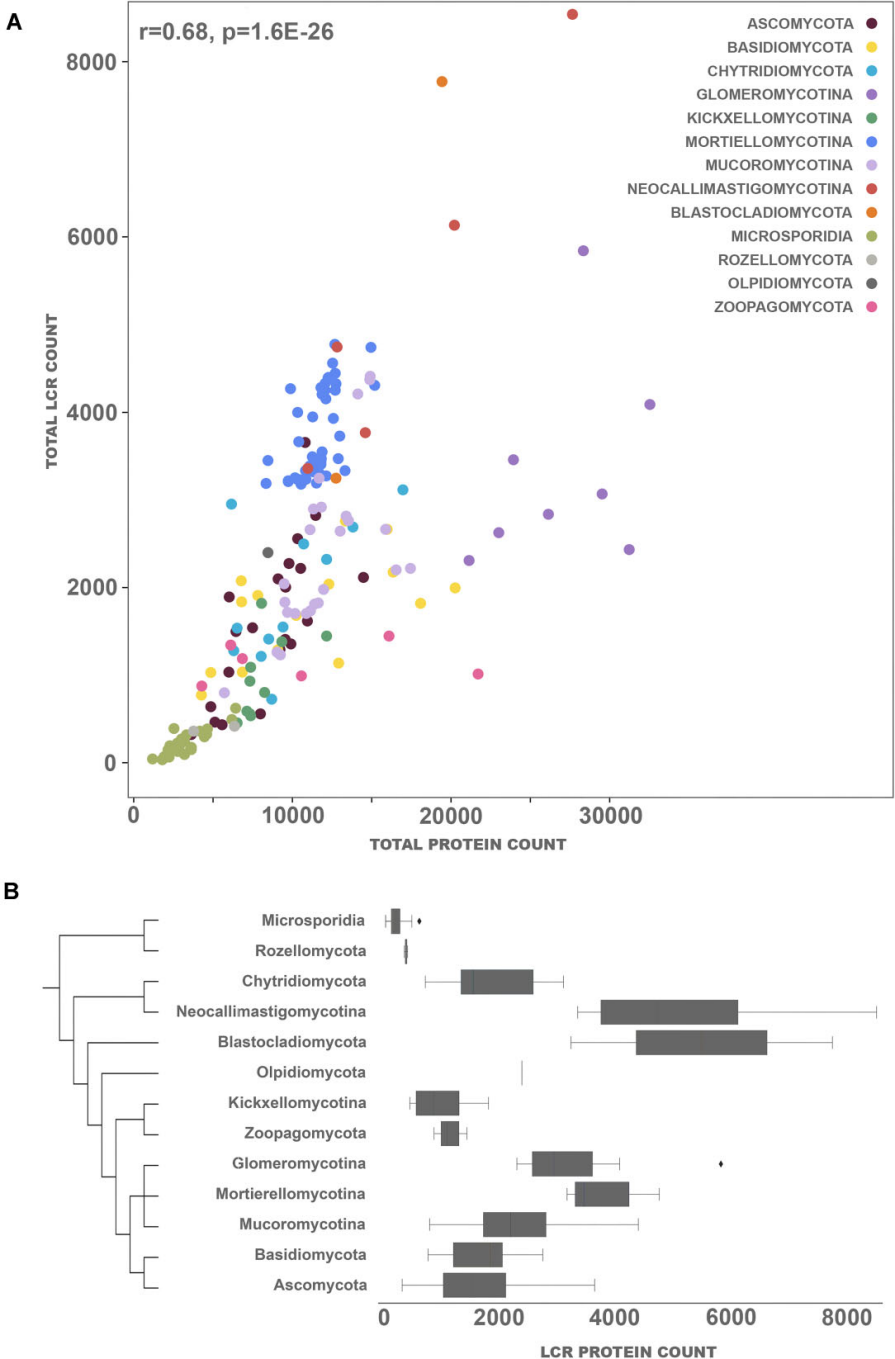
Total abundance of proteins with LCR varies among fungal phyla. (**A**) The abundance of LCR-containing proteins plotted against proteome size, colored by phylum with. (**B**) Overall abundance of proteins with LCRs per phylum. The taxonomic relationships between fungal lineages are derived from Spatafora *et al.* [40].

A second question which we addressed was the relationship between the number of LCRs and the size of the proteome. The overall abundance of LCRs is significantly correlated with the proteome size (*r* = 0.68) (Fig. 2A). Specifically, the number of domain-less with LCRs is more correlated with the overall proteome size (*r* = 0.77) compared to proteins with a domain and LCRs (*r* = 0.59) (Fig. 2). Particular fungal phyla differ in the abundance of proteins with LCRs (Fig. 2B). For instance, animal related Zoopagomycota and arbuscular mycorrhizal Glomeromycota tend to have fewer proteins with LCRs, and Mortierellomycota have more proteins with LCRs considering their proteome size. Moreover, Glomeromycota and parasitic Microsporidia have more often LCRs in proteins without domains than expected (Glomeromycota observed = 13 890, expected = 9785; Microsporidia observed = 3886, expected = 2295).

**Figure 3.**
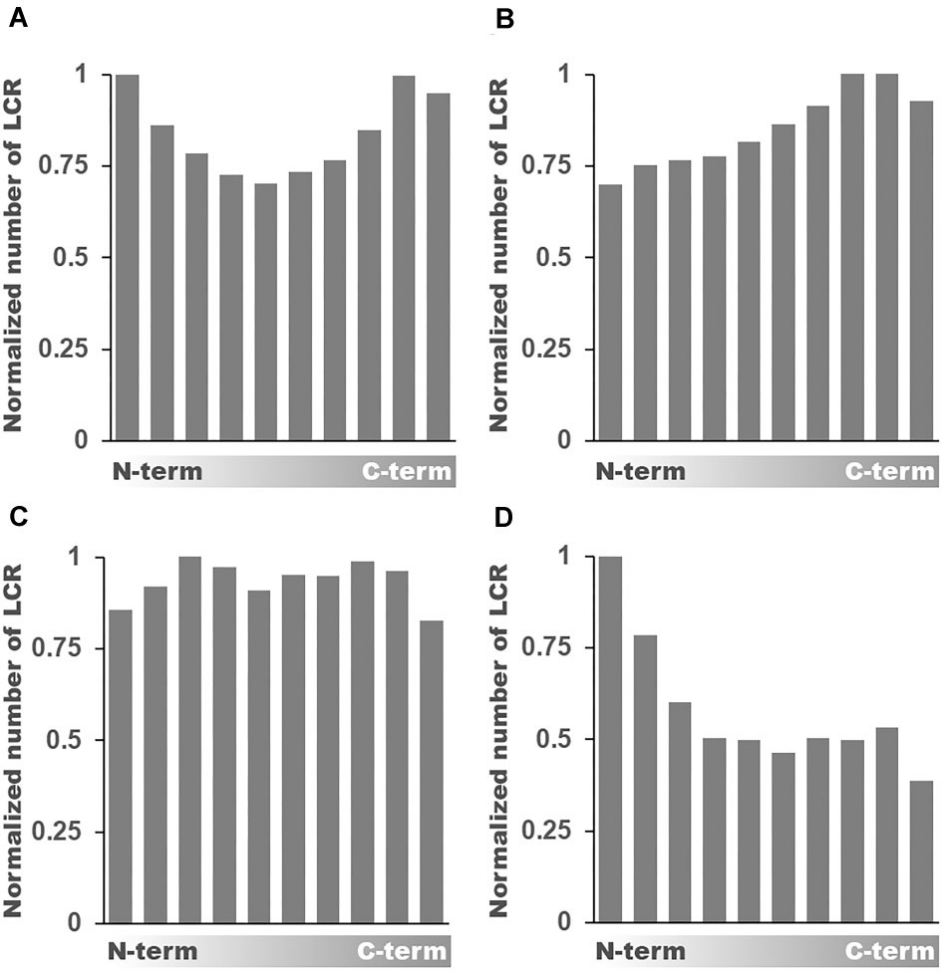
LCR localization within proteins is non uniform. Normalized distribution of LCR localization within the protein length treated as 10 bins starting from N- to C-terminus within all proteins (**A**), and specifically for Neocallimastigomycota (**B**), Microsporidia (**C**), and *Olpidium bornovanus* (**D**).

On average LCRs are located more commonly on either of the protein termini and are universally distributed across the central parts of the protein (Fig. 3A and Supplementary Fig. S9). However, there are exceptions to this rule with C-termini preference in anaerobic fungi Neocallimastigomycota (Fig. 3B), N-termini preference in the zoosporic parasite *Olpidium bornovanus*(Fig. 3D), as well as slight N- and C- termini depletion in Microsporidia (Fig. 3C). *O. bornovanus* genome assembly quality is limited (BUSCO score 27) and we consider this outlying preference for N-terminal LCRs as possibly influenced by the data. However, in the absence of other sequenced isolates we cannot rule out it being a real trait.

### Homopolymers constitute a small fraction of all LCRs and are less frequent than complex LCRs

**Figure 4.**
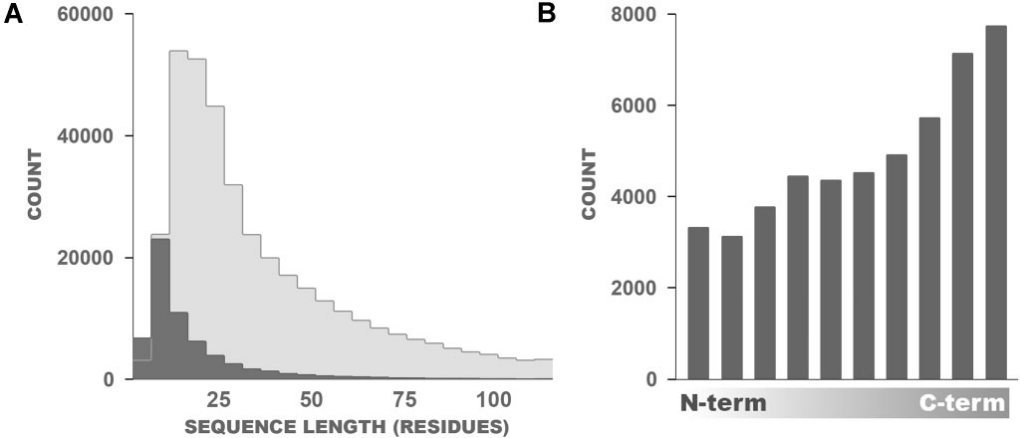
Simple LCRs composed predominantly of a single amino acid type are usually shorter comparing to the more complex LCRs (**A**) Distribution of lengths of homopolymers (dark gray) and complex LCR (light gray) as the number of amino acid residues. (**B**) For the relative localization of homopolymers within proteins, the length of individual protein is divided into 10 bins ranging from N- to C-terminus.

**Figure 5.**
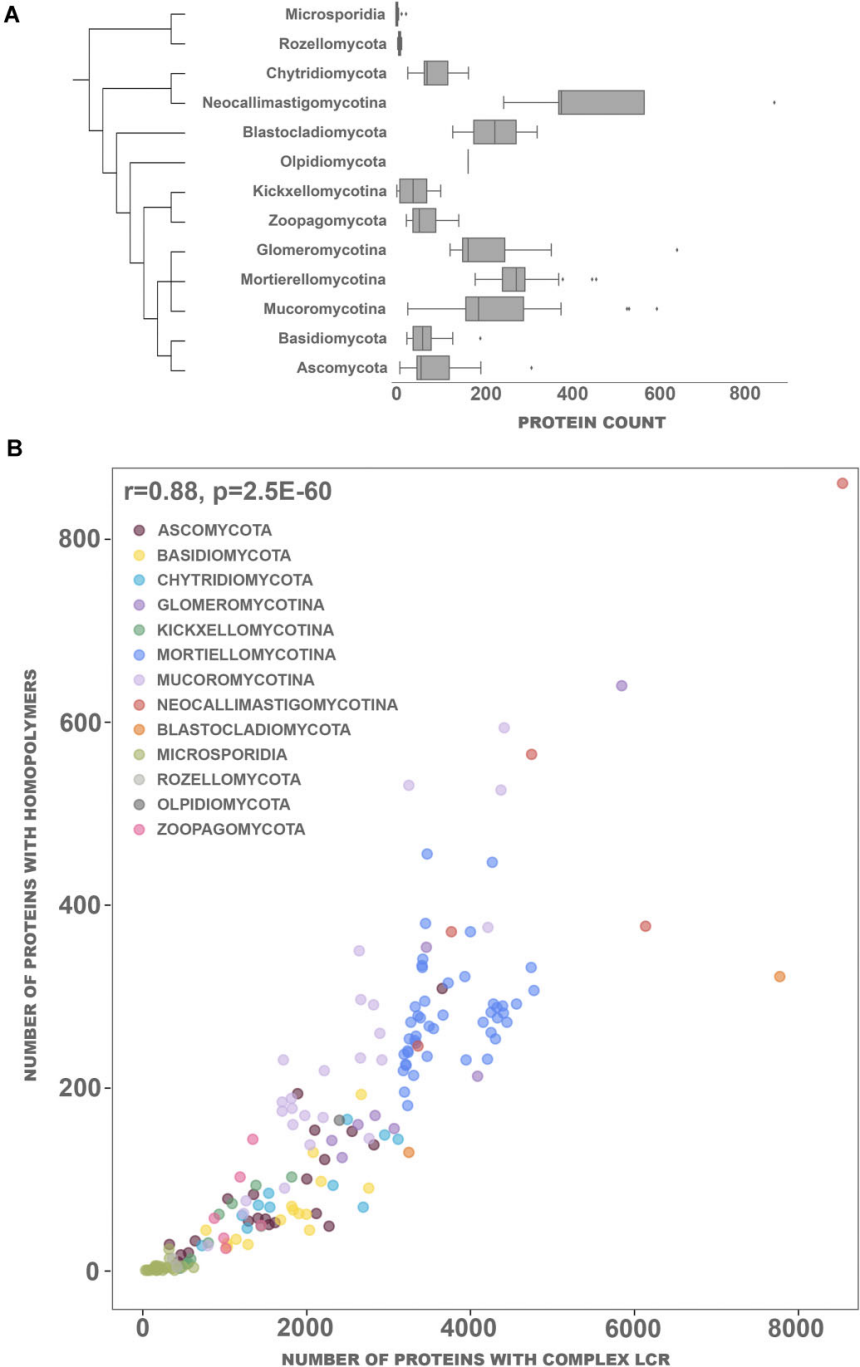
The number of proteins containing homopolymeric LCRs correlates with the total number of LCRs, and varies among fungal phyla. (**A**) Overall abundance of homopolymer-containing proteins per phylum. The taxonomic relationships between fungal lineages are derived from Spatafora *et al.* [40]. (**B**) Number of proteins with complex LCRs compared to the number of proteins with homopolymers per phylum.

When considering LCRs one can divide them into two categories, ones with a dominant type of amino acids referred to as homopolymers and LCRs with a more diverse amino acid composition referred hereafter as complex LCRs. Homopolymers are present in ∼6% of all the proteins with LCRs (28 664 of 401 761) and tend to be shorter than complex LCRs (10–20 versus 20–30 residues). Notably, 60% of the LCRs <10 amino acids are homopolymeric (Fig. 4A). Like all LCRs, homopolymers are located primarily on the protein termini, yet with clear preference for C-terminus (Fig. 4B). Homopolymers are most abundant in Neocallimastigomycota (median 377 homopolymers per proteome) and least in Microsporidia (median 3 homopolymers per proteome) (Fig. 5A). This difference can be explained to some degree by the extant correlation between proteome size and LCRs abundance. Moreover, there is a significant correlation between the abundance of LCRs and the number of homopolymers (*r* = 0.88) with a relatively stable share of homopolymer LCRs present in all taxa (Fig. 5B). Yet there are some deviations from this trend. For instance, Mortierellomycota have fewer homopolymers as expected from their number of LCRs (total number of homorepeats, *n* = 12 913; median of homorepeats in proteomes, *n* = 274; total LCRs count, *n* = 171 604) whereas Mucoromycota have relatively more homopolymers as of their overall LCRs abundance (*n* = 5906, *n* = 59 529) with moderate counts of homopolymers per proteome (median *n* = 189) (Fig. 5).

### LCRs rarely overlap with a protein domains

It is often disputed whether LCRs contribute to protein domains. We decided to inspect the relationship between the location of LCRs and presence of protein domains as well as the frequency of overlapping regions between LCRs and protein domains. We found that the overlapping pattern is not frequent and this preference applies to both complex and homopolymer LCRs. The share of complex LCRs overlapping with domains ranges from 3% (of all identified LCRs) in the proteome of *Olpidium bornovanus* to 10% in Rozellomycota, while LCRs distinct from domains account for 63% of all of the LCRs in Mucoromycotina to 30% in Microsporidia. All counts of the LCRs fractions: homopolymers, LCRs overlapping and nonoverlapping with domains, and LCRs in nondomain proteins are correlated with the proteome size and with the total abundance of LCRs (Supplementary Figs S2–S8).

**Figure 6.**
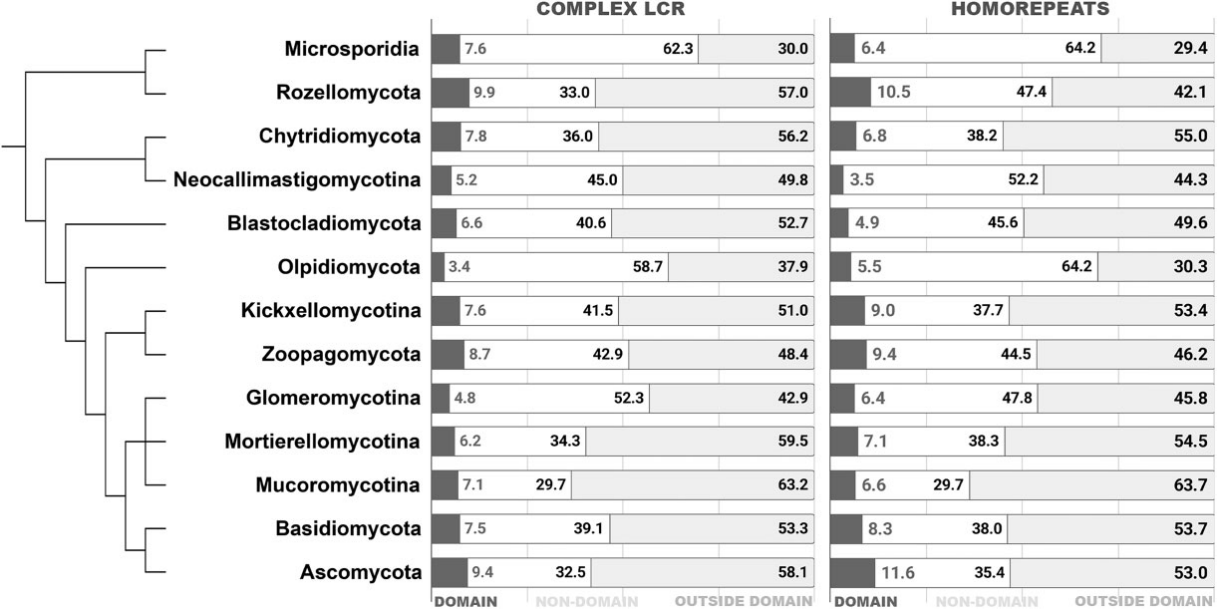
LCRs usually do not overlap with the domain regions in fungal proteins. Percentage of LCR localized “within” and “outside” of protein domains, as well as in nondomain proteins, for both complex LCR and homorepeats in multiple fungal phyla.

Similarly, homopolymers are also more often found in proteins with domains (61% of homopolymers) outside of the protein domains (89% of homopolymers). Homopolymer abundance varies across different phyla. Mucoromycotina has most of the homopolymers located in proteins with a domain but not overlapping the domain region (64%) whereas in Microsporidia they add up to only 29% of the homopolymers (Fig. 6). On the other hand Ascomycota has the highest percentage of homorepeats overlapping protein domains (12%), and Neocallimastigomycotina has the lowest at 4% (Fig. 6).

### One in three LCR is present in nondomain proteins

LCRs in nondomain proteins are most ubiquitous in Microsporidia (62% of all LCR therein) and least frequent in *Mucoromycotina* (30% of all LCR) (Fig. 5). The share of homopolymers in nondomain proteins varies from 64% in *Olpidium bornovanus* to 30% in Mucoromycotina (Fig. 6).

### Observed trends are not universal

In our analysis, we identified specific taxa that significantly diverge from the observed trends within the dataset, due to differing both proteome sizes and LCR numbers. *Allomyces macrogynus* ATCC 38327, a representative of the Blastocladiomycotina is a polyploid with a proteome size of 19447. *A. macrogynus* has 510 proteins with complex LCR overlapping with domains, 4295 proteins with LCR not overlapping domains and 3876 proteins without domains but with complex LCR. *Neocallimastix* sp. JGI-2020a, from Neocallimastigomycotina, also stood out with a proteome size of 27 675 and 425 proteins with complex LCRs overlapping with domains, in addition to 501 homopolymers present in proteins without domains. Pin molds, *Mucor plumbeus* and *Absidia repens*, (*Mucoromycotina*), have more than expected outside-domain homopolymers (*n* = 353, *n* = 330), to their respective proteomes (*n* = 11 690 and *n* = 14 915). *Absidia repens* further displayed 63 homopolymers within protein domains. Additionally, *Diversispora epigaea* (Glomeromycotina) and *Actinomortierella ambigua* (Mortierellomycotina) with contrasting proteome sizes of 28 348 and 9892, both exhibited the same number of 51 homopolymers overlapping protein domains. All of the deviations from dataset trends are visible in the Supplementary Figs S2–S8.

### LCRs often co-occur with specific protein domains

In order to draw functional links between LCRs and proteins we investigated which protein domains occur most often in proteins with LCRs (Table 1). These include RNA recognition motif, RRM_1 (PF00076) of which more than half contains an LCR, C-terminal domain of helicases, Helicase_C (PF00271) co-occurring with DEAD/H-box (PF00270) also enriched in LCR, protein kinase PKinase (PF00069)—the most common kinase associated with LCRs, and β-propeller repeat WD40 (PF00400). These protein domains are known to be present in multiple copies in many taxa, except for Microsporidia. All of the aforementioned LCR-containing proteins are expanded especially in Mortierellomycotina and Mucoromycotina. The amino acid composition of LCRs co-occurring with RRM_1, Helicase_C, PKinase, and WD40 domains are enriched in serine and glycine residues (each constitutes >13% of all amino acids in the LCRs).

**Table 1.** Protein domains most commonly accompanied by LCRs

Pfam acc.	Pfam ID	Comment (based on Pfam and InterPro)	Proteins with LCR	Expected
PF00076	RRM_1	RNA binding motif. Structure consists of four strands and two helices arranged in an alpha/beta sandwich	10 312	3879
PF00069	Pkinase	Catalytic domain of protein kinases. Found in serine/threonine tyrosine and dual specificity kinases	9882	6153
PF00096	zf-C2H2	Zinc finger domain. The two conserved cysteines and histidines coordinate a zinc ion	6328	2736
PF00271	Helicase_C	C-terminal domain in proteins belonging to helicase superfamilies 1 and 2. Included in this group is eukaryotic translation initiation factor 4A (eIF4A), member of the DEA(D/H)-box RNA helicase family. It has a parallel α,β-topology	5482	2643
PF00270	DEAD	DEAD and DEAH box helicases. Helicases are involved in unwinding nucleic acids	3837	1890
PF00806	PUF	Repeat domain made of two helices, PNecessary for sequence specific RNA binding in fly Pumilio and worm FBF-1 and FBF-2	3449	1218
PF00018	SH3_1	SH3 (Src homology 3) domains are often indicative of a protein involved in signal transduction related to cytoskeletal organization. The structure is a partly opened beta barrel	2894	994
PF00172	Zn_clus	N-terminal region of some fungal transcriptional regulators. Contains a Cys-rich motif involved in zinc-dependent binding of DNA. Forms binuclear Zn cluster, in which two Zn atoms are bound by six Cys residues	2726	1222
PF04082	Fungal_trans	Found in a number of fungal transcription factors including transcriptional activator xlnR, yeast regulatory protein GAL4, and nicotinate catabolism cluster-specific transcription factor HxnR	2277	993
PF00168	C2	Ca2+-dependent membrane-targeting module; important for signal transduction or membrane trafficking	2148	1050
PF00439	Bromodomain	Found in some DNA-binding proteins. Highly conserved α-helical motifs bind acetylated lysine residues on histone tails	2127	670
PF00320	GATA	Binds to DNA, uses four cysteine residues to coordinate a zinc ion. Two GATA zinc fingers build GATA transcription factors	2015	691
PF00226	DnaJ	Binds to dnaK (hsp70) and stimulates its ATPase activity	1965	949
PF05001	RNA_pol_Rpb1_R	Repetitive C-terminal domain of Rpb1 (RNA polymerase Pol II), plays a critical role in the regulation of gene expression	1964	442

Additionally, there are some phylum-specific peculiarities, most of them observed in two symbiotic phyla: *Neocallimastigomycota* and *Glomeromycotina*. In *Neocallimastigomycota*, there is a notable presence of LCR-containing proteins that have polysaccharide-binding domains and proteins with a β-propeller domain as well as Ankyrin repeats Ank_2 (PF12796). Polysaccharide-binding domains, specifically CBM_10 (carbohydrate-binding module 10, PF02013), CBM_1 (PF00734), and Chitin_bind_1 (PF00187), are rich in LCRs, with the majority of CBM_1 proteins containing LCRs. It has been shown that the CBM_10 family has been expanded in Neocallimastigomycotina’s cellulosomes as a protective measure against environmental factors [41]. Tyrosine constitutes >30% of these three polysaccharide-binding domains.

Glomeromycotina show an expansion of proteins with PK_Tyr_Ser-Thr (PF07714) domains, with more than half containing an LCR. In most taxa, Pkinase (PF00069) is the most common kinase associated with LCRs To add to that, Glomeromycotina stands out as the taxon with most LCRs in transposon-derived proteins, with domains such as Retrotrans_gag (PF03732), dUTPase (PF00692), Helicase_C (PF00271), and zf-CCHC (PF00098). There are on average 133 transposon-derived proteins with LCRs per proteome.

### Several classes of protein domains contain LCRs

To determine the possible contribution of LCRs to biological functions we analyzed the protein domains which include LCR regions. For instance enzymes such as Ubiquitin carboxyl-terminal hydrolase (UCH, PF00443; Petidase_C4 superfamily, CL0125) and NTPase AAA_11 (PF13086), transmembrane proteins such as 7tm_3 (PF00003), Cation_efflux (PF01545), MFS_1 (PF07690), and small HPS forming heterooligomeric aggregates HSP20 (Table 2). UCH peptidases and MFS_1 transporters with LCRs are widely spread across fungal taxa (on average *n* = 6 and *n* = 7 per proteome, respectively). LCR-containing 7tm_3 (PF00003) and HSP20 (PF00011) domains occur in high copy numbers in *Neocallimastigomycota*. Distinct fungal lineages have diverse LCRs in the same protein domains/protein families. The LCRs located in homologous domains may differ in location and composition. LCRs in 7tm_3 (PF00003) proteins are dominated by hydrophobic amino acids in *Neocallimastigomycota* these are mostly isoleucine, phenylalanine, leucine (I, F L; e.g. iailyfiisllillislfiiiif) whereas in Blastocladiomycota leucine, glycine, alanine, and isoleucine (e.g. lgllgalilaldalli). LCRs in UCH peptidases differ greatly between sequences even in the same organism with short and long, complex, and simple repeats with virtually any amino acid as the main component of the LCRs. LCRs seem to be gained and lost multiple times within a fungal order (as a figure Supplementary Fig. S10, as a tree file Supplementary File S10). For example, UCH peptidases KAG0297363.1 (LCRs mostly composed of S, T, and L) and KAG0297949.1 (LCR dominated by E, D, and K) originate from one fungus. Neocallimastigomycota is the phylum with the highest number of LCRs per genome and has only 5% of LCRs overlapping domains (for an abundance list of domains with LCR see Supplementary Table S1).

**Table 2.** Domains with LCRs inside

Pfam acc.	Pfam ID	Comment (based on Pfam and InterPro	Phylum	Overlap with LCRs
PF00428	Ribosomal_60s	This family includes archaebacterial L12, eukaryotic P0, P1, and P2	All	670
PF00443	UCH	Thiol proteases hydrolyzing peptide bonds at the C-terminal glycine of ubiquitin	All	437
PF01545	Cation_efflux	Integral membrane proteins—efflux pumps remove divalent metal ions like cadmium, zinc, and cobalt	All	313
PF07690	MFS_1	Transporters which transport small solutes in response to chemiosmosis	Almost all	278
PF00069	Pkinase	Catalytic domain of protein kinases. Found in serine/threonine, tyrosine and dual specificity kinases	Dikarya, Glomeromycota, Mortierellomycotina, Kickxellomycotina, Blastocladiomycota	195
PF02535	Zip	Zinc transport proteins and other metal transporters. Active in metal metabolism/homeostasis and widely involved in numerous physiological and pathological processes	Mortierellomycotina, Mucoromycotina, Microsporidia, Kickxellomycotina	170
PF00153	Mito_carr	Substrate carrier involved in energy transfer and found in the inner mitochondrial membrane or the membrane of other eukaryotic organelles, like peroxisome. Structurally, consists of up to three tandem repeats of a domain of ∼100 residues, each with two transmembrane helices	Mortierellomycotina, Chytridiomycota, Kickxellomycotina	149
PF13865	FoP_duplication	Conserved LDXXLDAYM region (where X is any amino acid), duplicated in the C terminus of chromatin target of PRMT1 protein (also known as Fop)	Mortierellomycotina, Chytridiomycota, Kickxellomycotina	126
PF00004	AAA	Large family of AAA ATPases (ATPases Associated with diverse cellular Activities), includes molecular chaperones, subunits of proteolytic complexes, independent proteases, DNA helicases or transcription factors	Mortierellomycotina, Chytridiomycota, Kickxellomycotina	113
PF00956	NAP	May act as histone chaperones, shuttling core and linker histones from their site of synthesis in the cytoplasm to the nucleus. May be involved in regulating gene expression	Mortierellomycotina	89
PF00003	7tm_3	C-terminal region of 3 GPCR receptors, containing seven transmembrane helices. These TM regions assemble to produce a docking pocket to which such molecules as cyclamate and lactisole can bind	Neocallimastigomycotina	82

There are several GO terms associated with PFAM domains significantly more abundant in proteins with LCRs (see Supplementary Table S1 for a complete list of GO terms together with the chi-squared values assigned to each category and Table 3 for the most diverged from expected). These include the nucleus, protein binding, DNA and RNA binding, and regulation of DNA-templated transcription with thousands of instances above expected values. One of the GO terms particularly enriched is cellulose binding with 729 proteins with LCR and 242 expected based on the number of all LCR containing proteins. This observation can be directly linked to the presence of LCRs in cellulosome components such as CBM proteins. Other substrate binding GO terms are also overrepresented in the set of LCR proteins including actin, ubiquitin, and phosphatidylinositol binding. On the other hand there are numerous categories depleted compared to the background, for instance, oxidoreductase activity, ribosome, transmembrane transporter activity, translation, proteasome, and proteolysis. Despite the common association of LCRs with repeat regions and nucleic acid processing the GO terms DNA integration is significantly underrepresented in the analyzed set of proteins. Moreover, binding of heme, iron, and pyridoxal phosphate binding are five times less frequent by LCR proteins than expected.

**Table 3.** GO categories with greatest deviations from the expected number of occurrences in proteins with LCRs

GO term	Protein count	Including proteins with LCRs	Expected for LCR proteins	Difference between observed and expected	Enrichment (LCR verss ALL proteins)
Structural constituent of ribosome	20 847	1288	5329	−4041	−19.38
Translation	20 478	1289	5234	−3945	−19.26
Ribosome	19 637	1202	5019	−3817	−19.44
Oxidoreductase activity	20 194	1657	5162	−3505	−17.36
Transmembrane transporter activity	21 227	2197	5426	−3229	−15.21
Catalytic activity	15 857	1728	4053	−2325	−14.66
Heme binding	9111	320	2329	−2009	−22.05
GTPase activity	12 561	1209	3211	−2002	−15.94
Iron ion binding	8888	412	2272	−1860	−20.93
Oxidoreductase activity, acting on paired donors	6983	151	1785	−1634	−23.4
Monooxygenase activity	6956	157	1778	−1621	−23.3
Biosynthetic process	6282	536	1606	−1070	−17.03
Pyridoxal phosphate binding	5095	298	1302	−1004	−19.71
Flavin adenine dinucleotide binding	4951	270	1266	−996	−20.12
Cellulose binding	949	729	243	486	51.21
GTPase activator activity	2053	1030	525	505	24.6
Phosphatidylinositol binding	3456	1436	883	553	16
Ubiquitin binding	1994	1095	510	585	29.34
Extracellular region	1865	1110	477	633	33.94
Actin binding	2534	1311	648	663	26.16
ATP-dependent chromatin remodeler activity	3528	1665	902	763	21.63
Protein dimerization activity	5595	2332	1430	902	16.12
Signal transduction	6555	2840	1676	1164	17.76
DNA-binding TF activity, RNA pol.II-specific	5893	2682	1506	1176	19.96
Sequence-specific DNA binding	5526	2839	1413	1426	25.81
Guanyl-nucleotide exchange factor activity	5507	2940	1408	1532	27.82
DNA-binding transcription factor activity	7409	3695	1894	1801	24.31
Nucleus	10 908	4918	2788	2130	19.53
RNA binding	26 072	10 825	6664	4161	15.96
Regulation of DNA-templated transcription	20 823	9833	5323	4510	21.66
Protein binding	98 107	30 654	25 077	5577	5.68

The analysis of functional categories can be generalized into a trend of LCR being more abundant than expected in proteins interacting with polymers such as nucleic acids and cellulose. On the other hand, proteins interacting with smaller molecules such as cofactors tend to have fewer LCRs than expected by chance.

### LCRs have a different amino acid composition from the proteomic background

**Figure 7.**
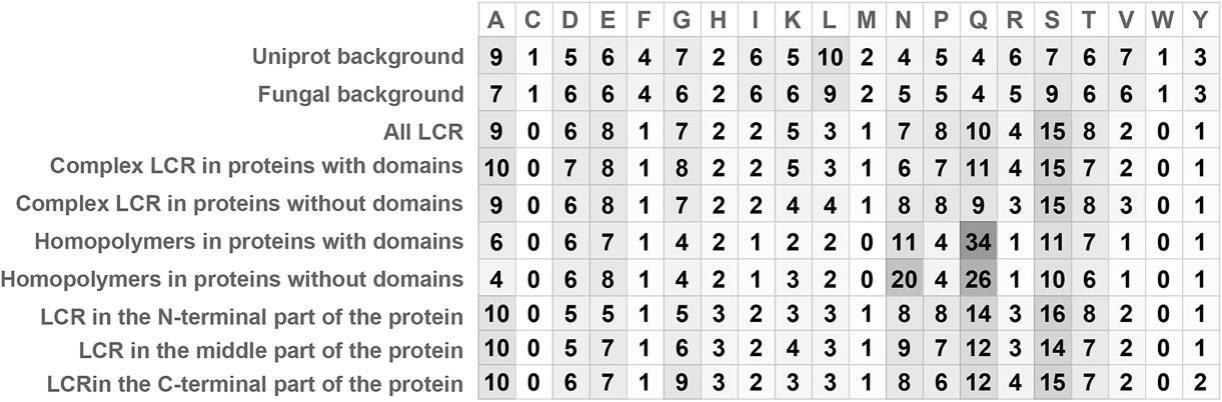
LCRs display a deviated amino acids composition compared to the UniProt. Average amino acid frequencies (as percentages) in LCRs, compared to the UniProt reference background.

Since LCRs are defined by their amino acid composition, we compared the frequencies of amino acids in the set of all fungal proteins and specific groups of LCRs. LCRs show biased amino acid composition compared to the UniProt frequencies and frequencies in the whole proteomes (considered as a background, Fig. 7). The overrepresented amino acids are A, D, E, P, N, Q, S, and T, so most of them have a functional group or are negatively charged. Underrepresented amino acids are mostly bulky aromatic and hydrophobic ones (C, F, I, L, M, V, W, andY). Particularly valine and leucine are depleted in LCR compared to the background. Also positively charged amino acids arginine and lysine are less commonly found in LCR relative to all of the proteins. LCR composition varies between fungal lineages but the overall trend with the leading abundance of serine and glutamine is conserved across taxa (Fig. 7). Some amino acids show a preference for LCRs at N- and C-terminal parts of the proteins. Particularly glycines and glutamic acids are often present towards the end of the proteins and less frequent at the N-terminus. Contrary to this, threonine and serine occur more often at the N-termini of proteins and less abundantly towards the ends.

Frequencies of amino acids in LCR are generally independent of the presence of domains in proteins; however, they differ between fungal phyla. We can observe high percentages of serine across all phyla except Olpidium, high glutamine contents in Mucoromycotina and Mortierellomycotina, as well as the biggest proportion of asparagine in Microsporidia and Neocallimastigomycota and a high content of alanine in Blastocladiomycota and Olpidium.

### Homopolymers stand out as a peculiar type of LCR

They show a different frequency of some of the amino-acids relative to both background proteome frequencies and observed amino acid frequency in complex LCR. On one hand, they show a 2- to 4-fold depletion of arginine, leucine, lysine, alanine, proline, and glycine compared to all of LCR sequences. On the other hand, they are enriched in glutamine (31%) compared to complex LCRs (10%), which makes glutamine the most abundant amino acid in homopolymers (Fig. 7). Similarly, we can observe a significant difference of asparagine frequency between complex LCRs (∼7%) and homopolymers (15%). In contrast, serine is less common in homopolymers than in complex LCRs. This trend, although with different proportions, can be observed for most phyla (Fig. 6). Poly-Q are more common in proteins with domains (34% residues versus 26% for nondomain proteins). Poly-N, on the other hand, seems to be more frequent in nondomain proteins (20% residues versus 11% in proteins with domains).

## Discussion

We present the first, wide-scale comparative study on LCRs within the whole taxonomic kingdom, pointing out novel, general observations that systematically support ideas formulated previously by other researchers working on LCRs from other organisms. LCR abundance is correlated with proteome size, but remains less dependent on genome size. This may suggest that LCR regions emerge stochastically within the proteins and the overall genome inflation driven mainly by DNA repeats and mobile elements [42] is not interfering significantly with the LCR content. LCR regions in fungi occur slightly more often in proteins with domains than in those without one, which is consistent with previous results showing notably higher amounts of LCRs within domain-containing proteins in mammals [43]. LCRs rarely overlap with the domain region, which may prevent interfering with the domain's function dependent on its amino acid and structural conservation [44]. As observed in yeasts, LCRs do not localize to the hydrophobic structural core of the protein [45]. Despite being generally unstructured, LCRs might attain secondary structures, with alpha-helix preference [46, 47], which might suggest their potential, yet minor roles within the overall protein structure.

LCR-containing proteins in fungi display significant enrichment in several GO terms, compared to non-LCR ones. Human LCR-containing proteins have shown enrichment in seven categories, the most numerous being “regulation of transcription” and “cell cycle” [13]. While in our dataset those categories are more prevalent in LCR-containing proteins, they are not as significantly enriched as other GO terms, like “nucleus,” ”protein binding,” ”regulation of DNA-templated transcription,” and “DNA binding.” These terms are connected to the canonical known LCR functions nucleic acid binding and protein–protein interactions [9, 45, 48, 49]. On the other hand, the most depleted categories of GO terms are “oxidoreductase activity” and “catalytic activity.” This seems to fit into general consensus that enzymatic activity, including oxidoreductase, is performed with the use of structurally conserved, nondisordered domains with specific residues for a given catalytic activity [50, 51]. Fungal homorepeats display similar GO terms distribution as complex LCRs, with enriched “nucleus,” “protein binding,” and “DNA binding,” but with the addition of “membrane,” which is significantly depleted in complex LCRs. This could point at preferred subcellular localization of homorepeat, as reported previously for eukaryotes and prokaryotes where homorepeats were shown to be the most enriched in proteins with nuclear or membrane localization [52].

LCRs in fungi show a preference towards protein termini, which has also been reported for yeast before [45]. However, preference for particular localization along protein’s sequence is not uniform but depends on organism and protein function. Proteins with LCR at either terminus perform mainly five functions: bind to RNA or DNA, influence gene expression [53, 54], facilitate protein–protein interactions [45, 48], enable proteins to adopt conformations necessary for processes like signal transduction [55] and enhance ligand binding specificity [9, 49]. It is very likely that fungal LCRs also follow this trend.

Amino acid composition of low-complexity regions has been analyzed thoroughly in prokaryotes [9]. Our results are consistent with some of the observations reported by Ntountoumi *et al.* For instance, the reduced occurrence of hydrophobic and aromatic amino acids seems to be a universal feature of LCR regions which do not disrupt protein's structural core but rather are exposed for interaction with other molecules. These observations support the hypothesis on the involvement of LCR regions in protein-protein interactions and regulatory functions raised for instance by Lee *et al.* [56]. Moreover, protein interactions or posttranslational modifications require the functional groups of e.g. serines and threonines, overrepresented in LCR regions. In prokaryotes, LCR regions were often identified in proteins related to translation, nucleic acid, ion and polysaccharide binding. Moreover, particular functions were correlated with specific LCR compositions. For instance, in the study by Ntountoumi *et al.* [9], bacterial proteins with assigned GO category “carbohydrate binding” had LCR rich in serines and threonines—amino acids frequent also in fungal LCR. After all, carbohydrate binding modules are paramount for fungal intracellular metabolism and nutrient acquisition. Serine/arginine-rich proteins have been shown to undergo conformational switches when phosphorylated [57]. Although bacterial serine-rich proteins have no obvious regulatory mechanism [58], in fungi, poly-serine regions are likely to be O-glycosylated [59]. In contrast to the observed abundance of positively charged amino acids in prokaryotes [9], fungal LCRs were depleted in arginine and lysine. The relative scarcity of the aforementioned amino acids likely results in a diminished ability to bind negatively charged molecules like nucleic acids and fatty acids. Perhaps this relates to the compartmentalization which screens DNA from cytoplasmic proteins.

Glomeromycota and microsporidia have fewer LCR than expected from their proteome sizes, which is visible both for proteins with and without domains. This is particularly intriguing considering the overall repetitive content of Glomeromycotina genomes and proteomes [60]. On the other hand, Mortierellomycota are extraordinarily rich in LCR, which are mostly present in expanded protein families. LCR abundance seems to be at least partially driven by the accumulation of duplications of protein families, most often including Pkinase, RRM, WD40, Helicase_C, and Ank-containing proteins—domains massively expanded in fungi [61].

Pkinase, RRM, WD40, and Helicase_C domains are associated with LCR in most fungal phyla. There are known differences between kinase families in fungi, which primarily lie in their functional adaptations and stress–response mechanisms. Pkinase domains are the most common kinase family in the fungal kingdom, integrating a variety of signals that synchronize key processes of the fungal life cycle [62], such as hyphal growth, biofilm formation, and pathogenicity, the latter well described in *C. neoformans*, in which those domains were the most commonly associated with virulence associated factors [63]. Pkinases are especially expanded in *Rhizopus* spp. [64]. This domain is often encountered at the N-termini of NOD-like receptors [65]. One might speculate on the contribution of LCR to protein–protein interactions of kinases or the phosphorylation of kinases because these LCRs are rich in serine [45, 66].

RRM is the most common RNA binding motif in fungi and is present in proteins involved in all the steps of RNA metabolism [67]. The interaction of RRM with RNA is particularly influenced by the boundary residues at the C-terminus of RRM as has been shown for Fused in Sarcoma proteins where RRM boundary connects with neighboring disordered region, facilitating RNA binding [45, 68].

WD40 are recognized as network-building proteins [69, 70]. For WD40 domains, the overlapping low-complexity sequences between repeats, such as the glycine-rich track found in proteins of protozoan parasite *Plasmodium vivax*, suggest a structural or functional role despite the lack of clear sequence conservation. These repeats, exposed on the domain’s bottom face, may contribute to its interactivity or stability, though their exact significance remains to be fully understood [71].

Helicase_C domains are often co-opted from fungal transposons [72], but the relationship between LCR and transposable elements is unknown. On one hand, regions of DNA repeats could contribute to protein sequence repeats; on the other, the presence of LCR might enhance the connection of the domesticated transposase to the protein network of the host. However, the relationship between DNA repeats and protein repeats is weak at least in model eukaryotes [73]. The ubiquity of transposable elements in Glomeromycota genomes is well documented and perhaps creates a sequence space for LCR proliferation. We observed TE-related domains among the most ubiquituous in LCR-containing proteins of this phylum. One of the most common TE-related domains in fungi is Helicase_C. The presence of DEAD/H-box domains alongside Helicase_C domains, often with disordered regions, may be necessary to shape protein’s binding specificity [74, 75]. Consistently, all of the aforementioned functionalities have been listed along with other LCRs properties [9, 45].

Insertions of LCR into protein domains are not very common but not negligible. Particularly enzymatic domains rarely overlap with LCR. One such example is the UCH exopeptidases, responsible for the hydrolysis of C-terminal glycine of ubiquitin. Half of the LCR containing UCH peptidases are found in Mortierellomycotina. So far the insertion of LCR into enzymatic domains has been documented for Tet dioxygenases catalytic region, negatively regulating this enzyme’s activity [76] and orotidine 5′-monophosphate decarboxylase in *Plasmodium falciparum*. In this case, LCR is responsible for this enzyme’s interaction with orotate phosphoribosyltransferase, crucial components in the pyrimidine biosynthetic pathway [77]. However, the possible impact of the LCR on the enzymatic function remains unknown.

Distribution of homopolymers differs between phyla. Homopolymers are more numerous in symbiotic Glomeromycota and rumen-specific Neocallimastigomycota than in other phyla. This richness can be explained by the large overall proteome sizes of these fungi. The abundance of homopolymers LCRs in cellulosome components opens up the question on the role of LCR in adaptability. We noticed elevated numbers of LCRs in proteins with the cellulose binding CBM_10 domain. These proteins are bacterial xenologs with common domain fusions post-transfer [78]. The presence of poly-N tracts in Neocallimastigomycota and, to a lesser degree, in Microsporia may lead to the formation of unique structures due to interactions of the asparagine side chains. Mucoromycotina possess more homopolymers than expected from their moderate proteome sizes. These homopolymers are most often polyQ tracts. PolyN and PolyQ tracts are linked to prion formation [79, 80]. PolyA found among others in Blastocladiomycota were described in animals as prone to form α helices present in signal peptides and mitochondrial transit peptides [81].

Proteomic flexibility could confer advantages in host interaction or evasion of host defences and co-occurring organisms [82]. Homopolymer LCRs tend to be shorter than complex LCRs, with their highest proportion falling in the range of less than 15 amino acids. Short homopolymer tracts have been reported before and can be explained by protein structural constraints and tradeoffs related to polymerase slippage and folding difficulties [83]. Amino acid composition of homopolymers varies across fungal phyla, but the most equal distribution across all fungal phyla has been found for homorepeats of polyS and polyQ. Those two types of homopolymers are described as being generally the most abundant types of repeats in all taxa [84, 85].

Studies in fungi have provoked major advancements in cell biology. After all, it is yeast which has been the first sequenced eukaryote, and still remains the model eukaryotic organism. However, model species itself obviously cannot reflect the diversity of the tree of life, so that a more comprehensive set of organisms is needed to infer evolutionary patterns. Our attempt to assess the diversity of LCRs across a eukaryotic kingdom can serve as a comparative resource for both prokaryotic and eukaryotic studies. Fungal proteomes are usually more compact compared to plants and animals, and in general have fewer repeat regions. In fact, they are sometimes comparable to bacterial proteomes. On one hand, similarities in LCRs amino acid composition reported in most of the previous LCR studies (polyQ and polyN) suggest an eminent role of LCRs in polymer binding and protein-protein interactions. On the other hand, differences between fungal LCRs and bacterial ones (no enrichment in positively charged amino acids in fungal LCR) point at distinct evolutionary pathways in organisms with a nucleus compartment and without it.

We observed that proteins with domains involved in interactions with complex molecules such as DNA, RNA, phospholipids, and cellulose tend to have more LCRs than expected by chance. This might suggest that their polymer binding function benefits from extensive and unstructured tracts of polar residues for general interaction with macromolecules of a given kind. On the contrary, proteins with domains binding small molecules such as cofactors tend to be depleted in LCRs. Such proteins would rather rely on a precisely defined set of interacting partners, so that introduction of unstructured regions might disturb their potency for proper interactions and functions.

At more general, GO terms level, the very core translation (structural constituent of ribosome, translation, and ribosome), respiration (oxidoreductase activity, heme binding, iron ion binding, monooxygenase activity, FAD and NAD binding, and proton transmembrane transport), proteolysis (proteasome core complex and proteolysis involved in protein catabolic process), enzymes (catalytic activity), and transmembrane transport (transmembrane transporter activity and proton transmembrane transport) require proteins depleted in LCR. On the other hand, nucleic acids binding (DNA-binding transcription factor activity, RNA polymerase II-specific, sequence-specific DNA binding, DNA-binding transcription factor activity, RNA binding, regulation of DNA-templated transcription, rRNA processing, ATP-dependent chromatin remodeler activity, and helicase activity) and functionally connected zinc binding (zinc ion binding) as well as nuclear localization (nucleus) are more abundant in LCR. So are proteins involved in interactions with actin (actin binding, protein–protein interactions, and protein dimerization activity) and cellulose (extracellular region and cellulose binding). Nucleic acid binding, although also at the very core of life, often requires extensive, nonspecific interactions with negatively charged polyphosphate backbones. Hence, the distribution of LCR emerges stochastically and after fixation seems to support the functional foundations of the proteome.

## Supplementary Material

lqaf014_Supplemental_Files

## Data Availability

The dataset(s) supporting the results of this article is available in the Zenodo repository https://zenodo.org/records/13928873.
