# Supplementary material for: Low-complexity regions in fungi display functional groups and are depleted in positively charged amino acids
Source: NAR Genom Bioinform. 2025 Feb 27;7(1):lqaf014. doi: 10.1093/nargab/lqaf014 (PMC11878562; doi:10.1093/nargab/lqaf014)
Supplement: lqaf014_Supplemental_Files [file lqaf014_supplemental_files.zip › Supplementary Figures SF1.pdf]

Low complexity regions in fungi display functional groups and are depleted in positively charged amino-acids

Kamil Steczkiewicz[1], Aleksander Kossakowski[1], Stanisław Janik[1,2] and Anna Muszewska*[1]

[1] Institute of Biochemistry and Biophysics, Polish Academy of Sciences, Pawinskiego 5A, 02-106 Warsaw, Poland
[2] Faculty of Mathematics, Informatics and Mechanics, University of Warsaw, Stefana Banacha 2, 02-097 Warsaw, Poland

LIST OF SUPPLEMENTARY FIGURES

Supplementary Figure S1 - Correlation map for protein features. 2

Supplementary Figure S2 - Correlation between the number of proteins with non-domain homopolymer LCRs and total proteome size colored by phylum 3

Supplementary Figure S3 - Correlation between the number of proteins with homopolymer LCRs overlapping with domains and total proteome size colored by phylum 4

Supplementary Figure S4 - Correlation between the number of proteins with homopolymer LCRs not overlapping with domains and total proteome size colored by phylum 5

Supplementary Figure S5 - Correlation between the number of proteins with LCRs overlapping with domains and total proteome size colored by phylum 6

Supplementary Figure S6 - Correlation betweenthe number of proteins with complex LCRs not overlapping a domains and total proteome size colored by phylum 7

Supplementary Figure S7 - Correlation between the number of non-domain proteins with complex LCRs and total proteome size colored by phylum 8

Supplementary Figure S8 - Correlation between the number of proteins with homopolymer LCRs and total proteome size colored by phylum 9

Supplementary Figure S9 - Relative LCR position (protein length normalized to 10) in proteins of A) Mortierellomycotina B) Zoopagomycota C) Rozellomycota D) Glomeromycotina E) Kickxellomycotina F) Ascomycota G) Chytridiomycota H) Basidiomycota I) Mucoromycotina and J) Blastocladiomycota. 10

Supplementary Figure S10 - ML tree computed with Iqtree2 showing the distribution of LCR in UCH peptidases across Mucoromycotina representatives. 11

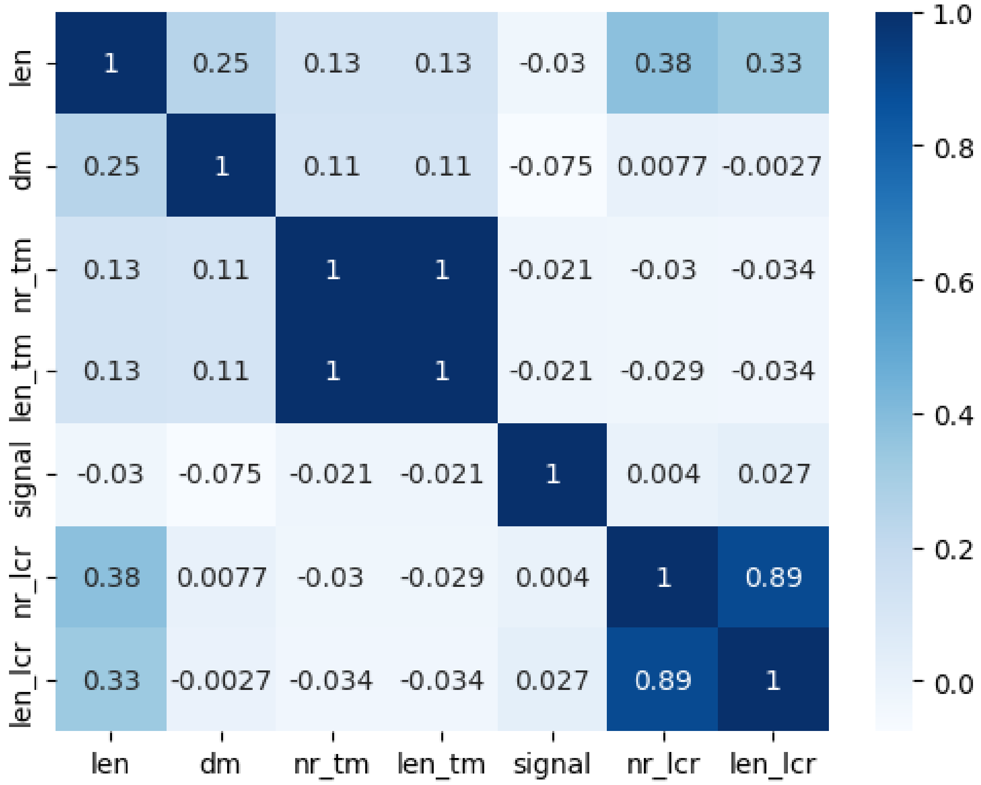

Supplementary Figure S1 - Correlation map for protein features.

The table shows lack of interdependence between the presence or length of LCRs and other protein attributes such as presence of domains (dm), length of the protein (len), number of transmembranes (nr_tm), length of transmembranes (len_tm) and presence of a signal sequence (signal).

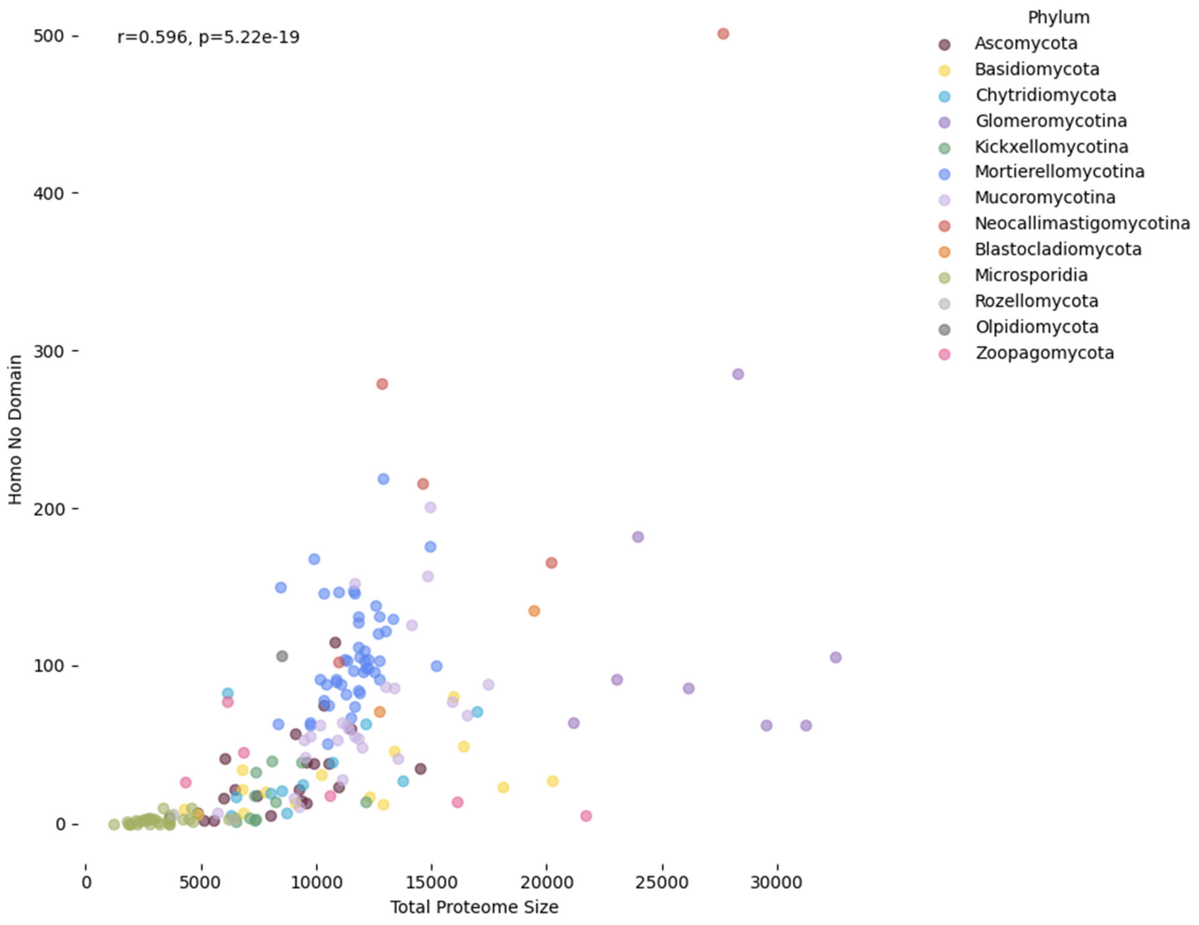

Supplementary Figure S2 - Correlation between the number of proteins with non-domain homopolymer LCRs and total proteome size colored by phylum

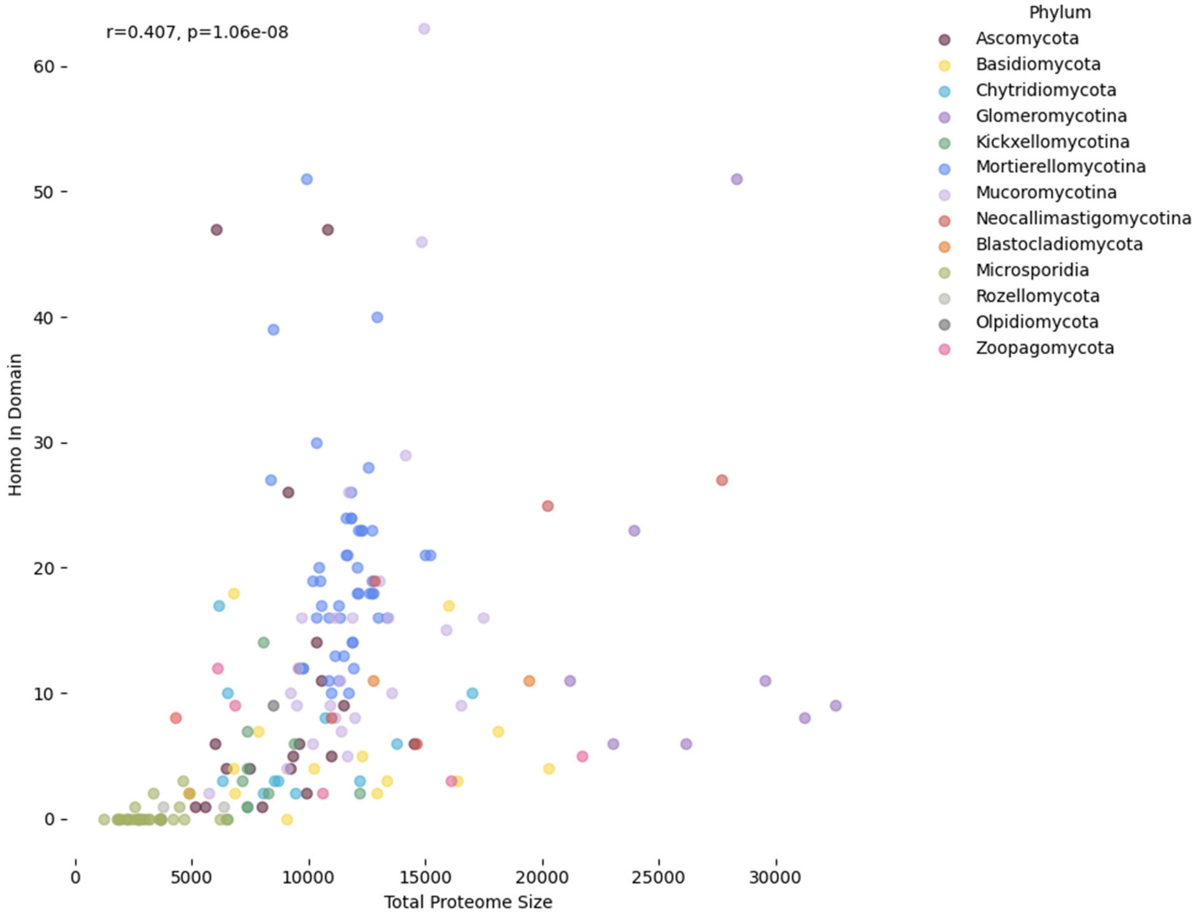

Supplementary Figure S3 - Correlation between the number of proteins with homopolymer LCRs overlapping with domains and total proteome size colored by phylum

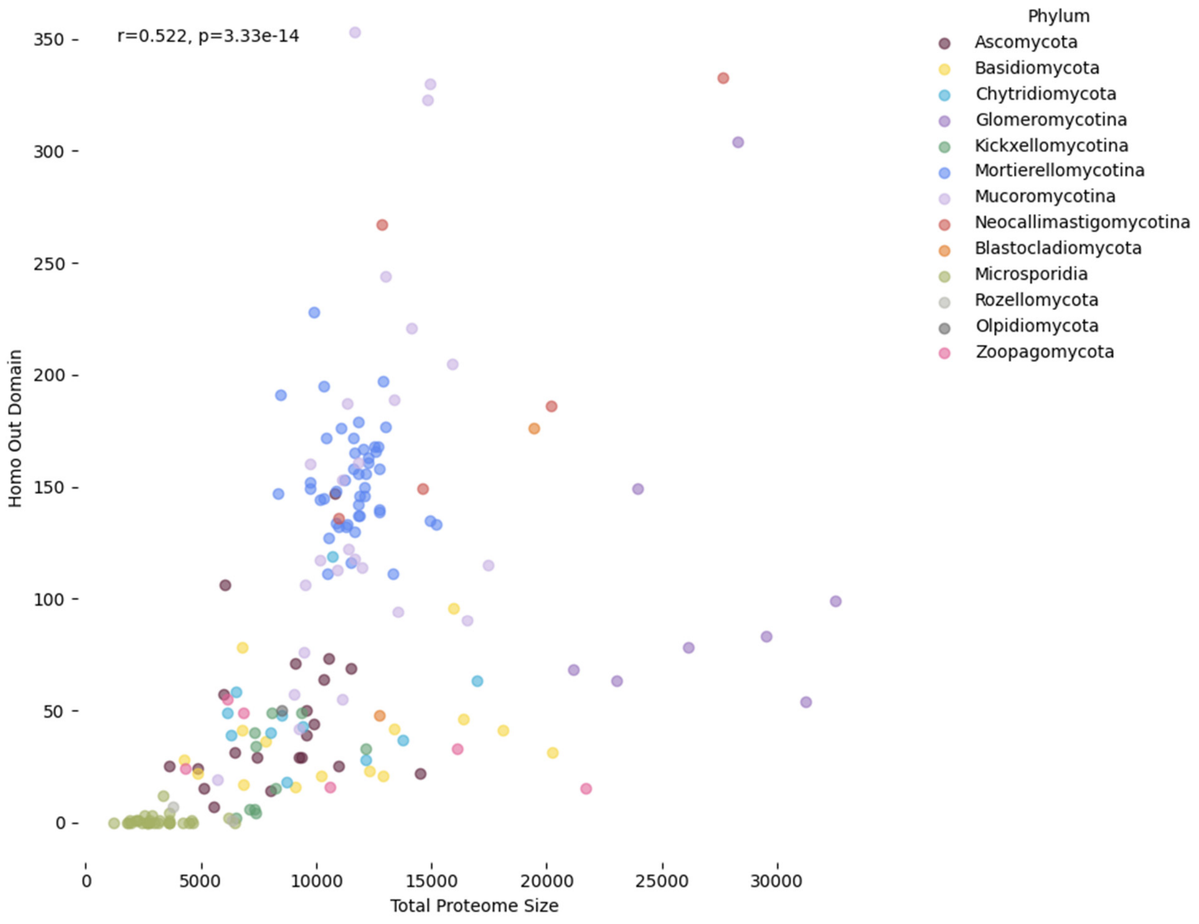

Supplementary Figure S4 - Correlation between the number of proteins with homopolymer LCRs not overlapping with domains and total proteome size colored by phylum

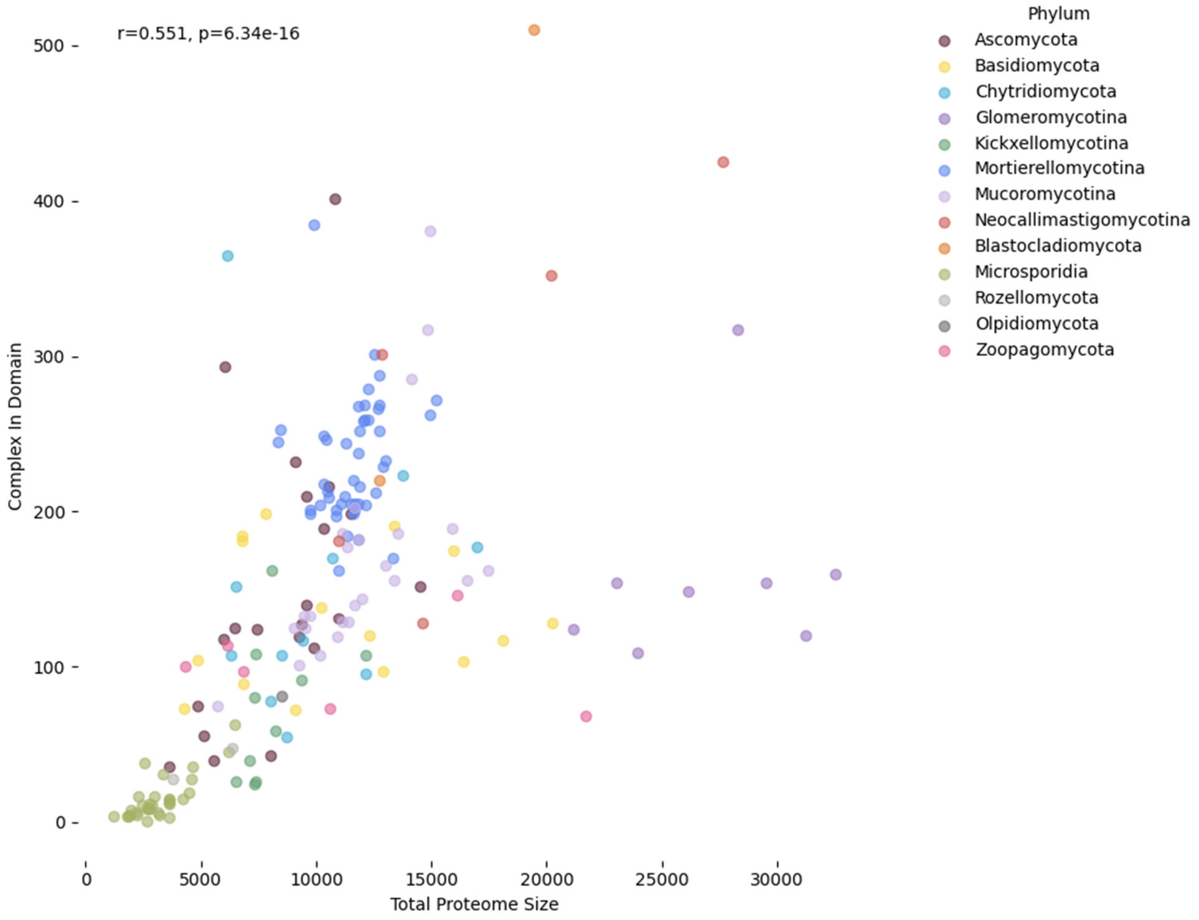

Supplementary Figure S5 - Correlation between the number of proteins with complex LCRs overlapping with domains and total proteome size colored by phylum

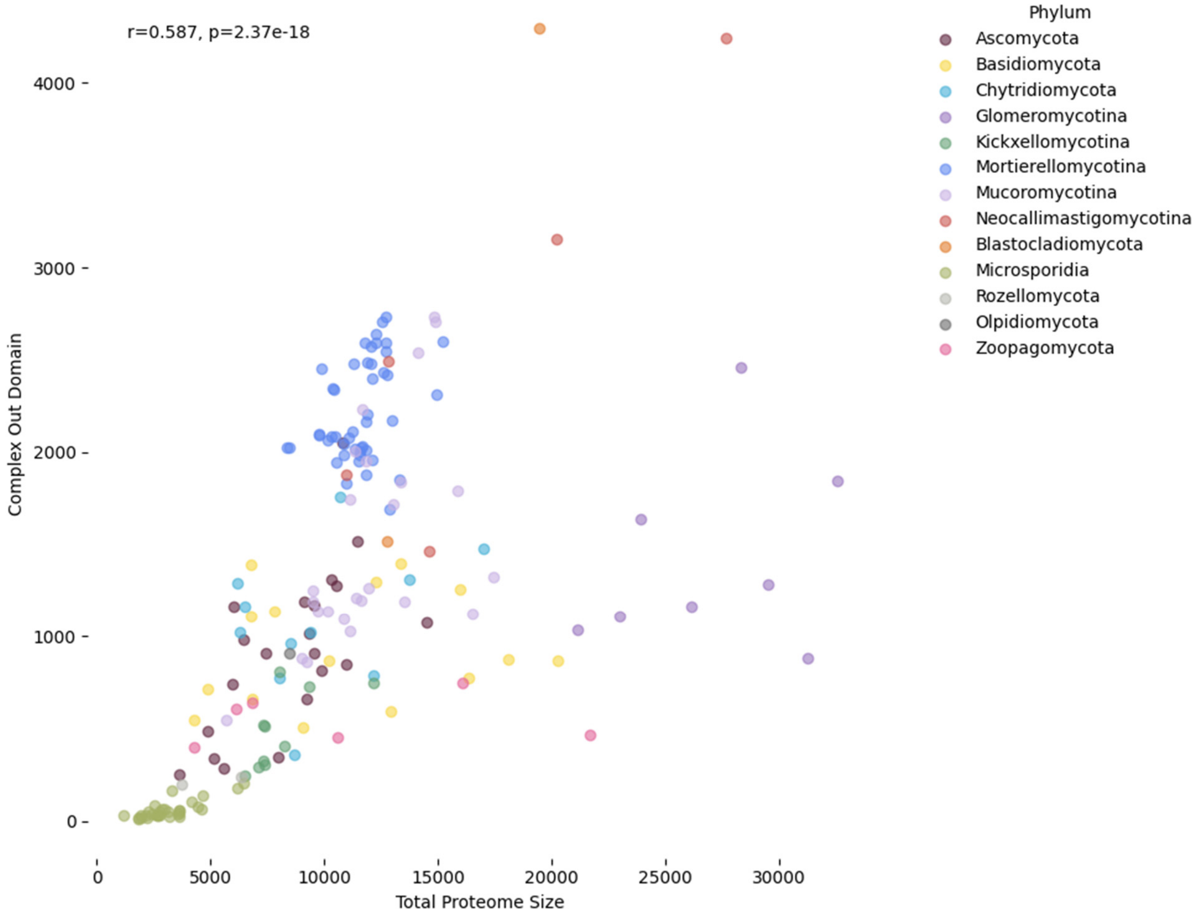

Supplementary Figure S6 - Correlation between the number of proteins with complex LCRs not overlapping a domains and total proteome size colored by phylum

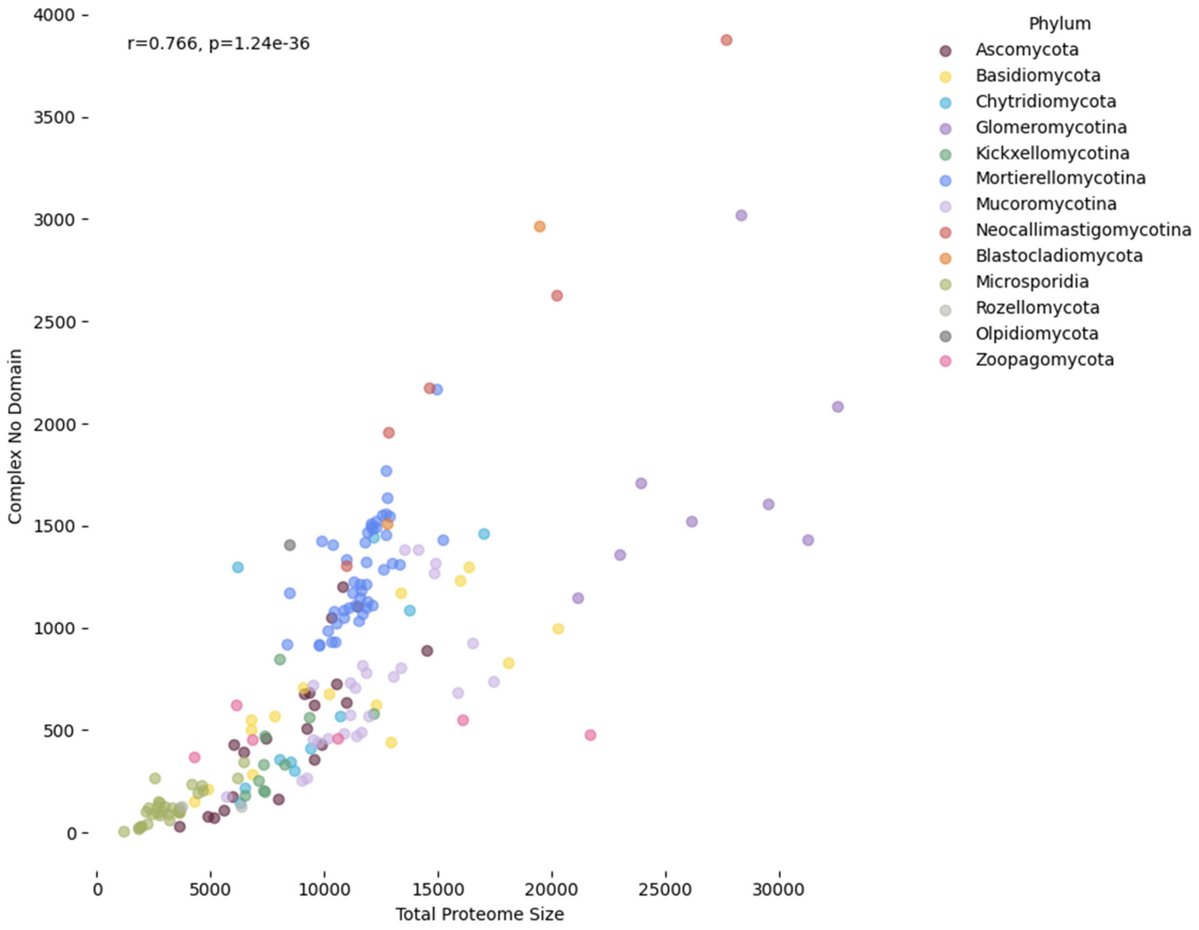

Supplementary Figure S7 - Correlation between the number of non-domain proteins with complex LCRs and total proteome size colored by phylum

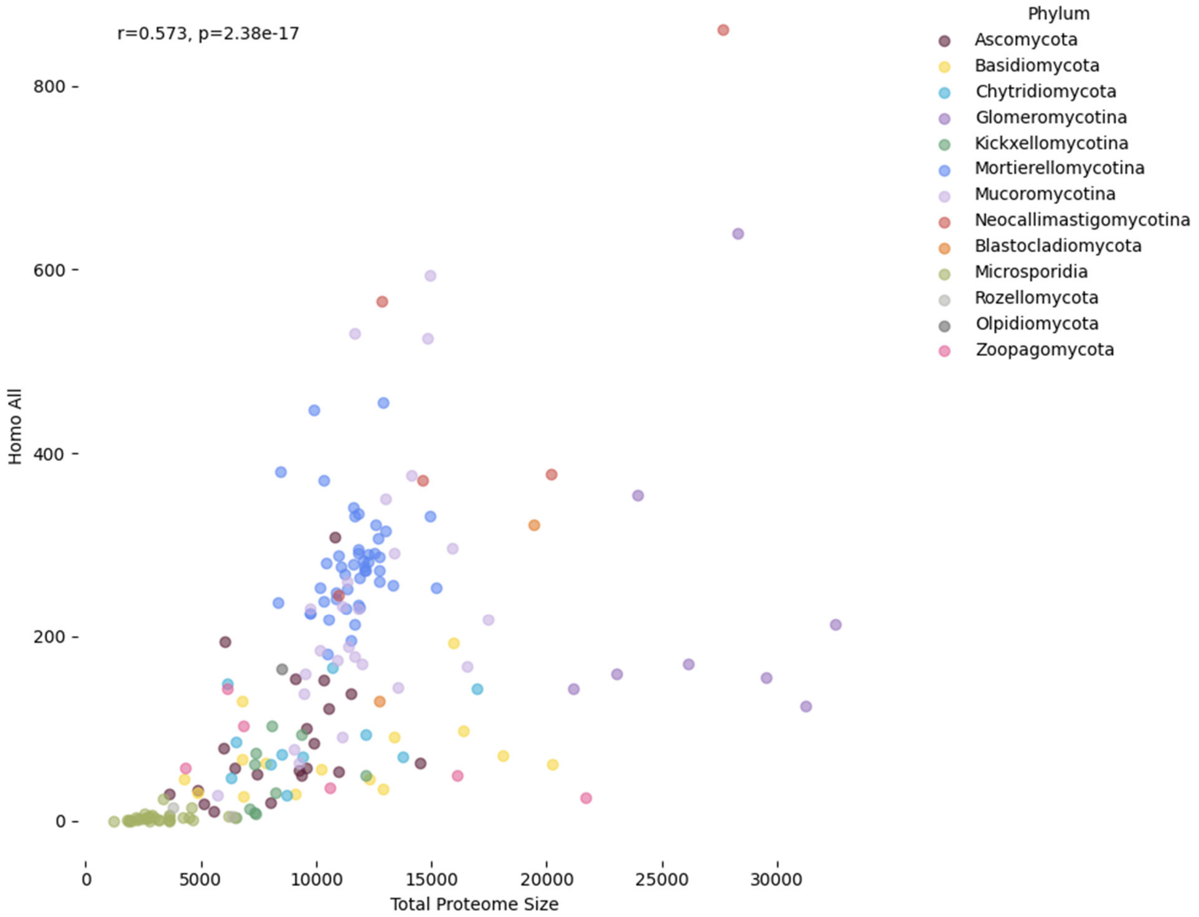

Supplementary Figure S8 - Correlation between the number of proteins with homopolymer LCRs and total proteome size colored by phylum

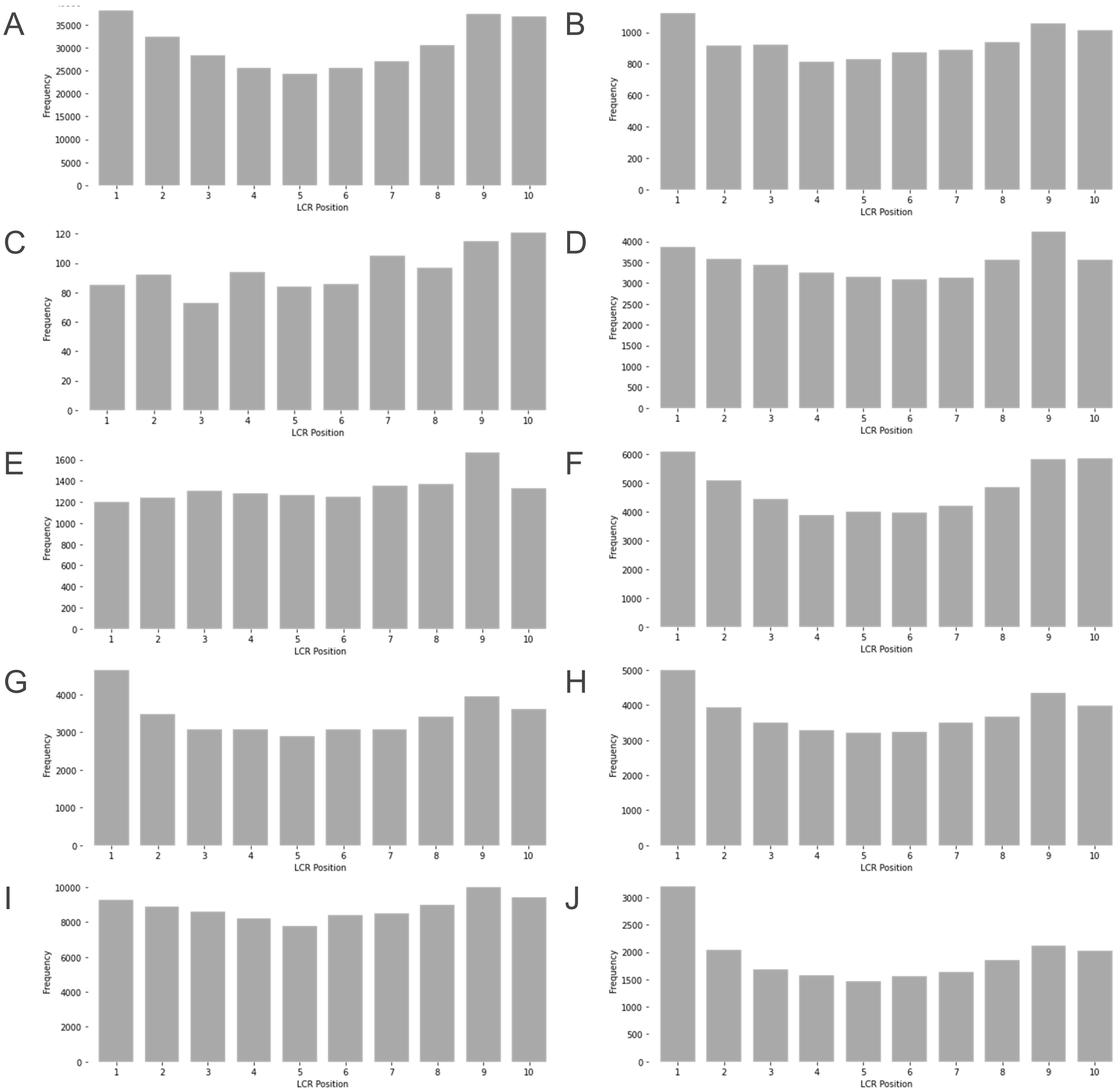

Supplementary Figure S9 - Relative LCR position (protein length normalized to 10) in proteins of A) Mortierellomycotina B) Zoopagomycota C) Rozellomycota D) Glomeromycotina E) Kickxellomycotina F) Ascomycota G) Chytridiomycota H) Basidiomycota I) Mucoromycotina and J) Blastocladiomycota.

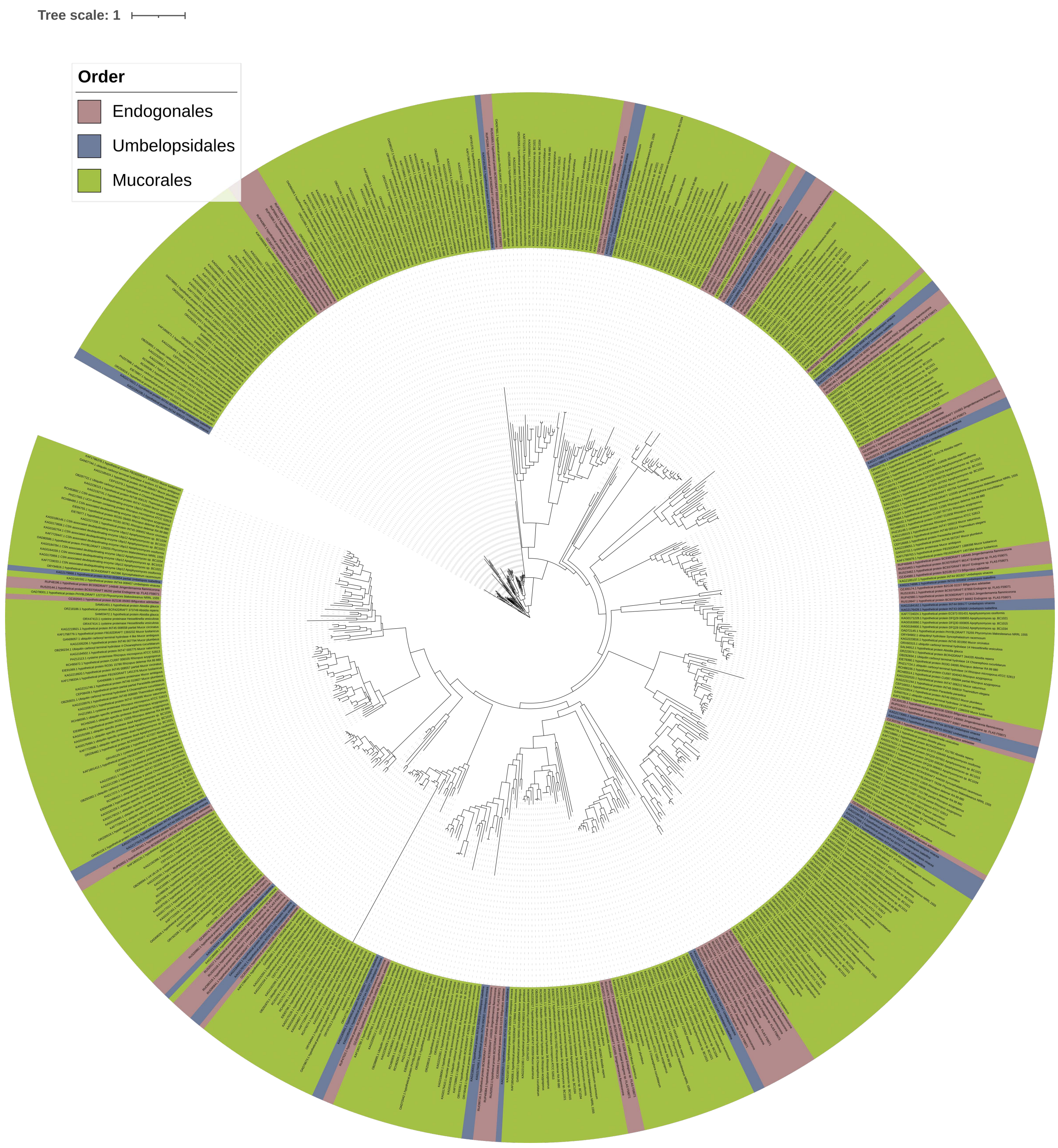

Supplementary Figure S10 - ML tree computed with Iqtree2 showing the distribution of LCR in UCH peptidases across Mucoromycotina representatives.

Orange circles denote LCRs nonoverlapping the UCH domain, dark purple squares - LCRs in UCH domains. The phylogenetic tree was inferred for all protein sequences of Mucoromycotina UCH peptidases present in the dataset.
