# Supplementary material for: Low-complexity regions in fungi display functional groups and are depleted in positively charged amino acids
Source: NAR Genom Bioinform. 2025 Feb 27;7(1):lqaf014. doi: 10.1093/nargab/lqaf014 (PMC11878562; doi:10.1093/nargab/lqaf014)
Supplement: lqaf014_Supplemental_Files [file lqaf014_supplemental_files.zip › Supplementary.docx]

**Supplementary Table S1.** stores NCBI identifiers of genomic assemblies with taxonomic assignment and original publication references (sheet Dataset_organisms); GO terms inference from pfam2go for all domains found together with LCRs (sheet GO_PFAM36); all PFAM domains found in proteins with LCRs (sheet pfam_enrichment with chi-squared test values and pfam_changes with descriptions of the domains obtained from a MySQL pfam database); Pfam domains distribution in phyla for domains neighboring complex LCRs (sheet complexLCR_outside_domain), for domains with LCR insertions (sheet complexLCR_inside_domain) for domains neighboring homopolymers (sheet homopolymerLCR_outside_domain) and domains with homopolymer insertions (sheet homopolymerLCR_inside_domain).

**Supplementary File S2.** Contains a perl script to integrate results from sequence analyses and a Jupyter notebook with all commands needed to draw figures based on the dataset.

**Supplementary Figures** - Supplementary materials are available at NAR online
