# Supplementary material for: Low-complexity regions in fungi display functional groups and are depleted in positively charged amino acids
Source: NAR Genom Bioinform. 2025 Feb 27;7(1):lqaf014. doi: 10.1093/nargab/lqaf014 (PMC11878562; doi:10.1093/nargab/lqaf014)
Supplement: lqaf014_Supplemental_Files [file S10.pdf › S10.pdf]

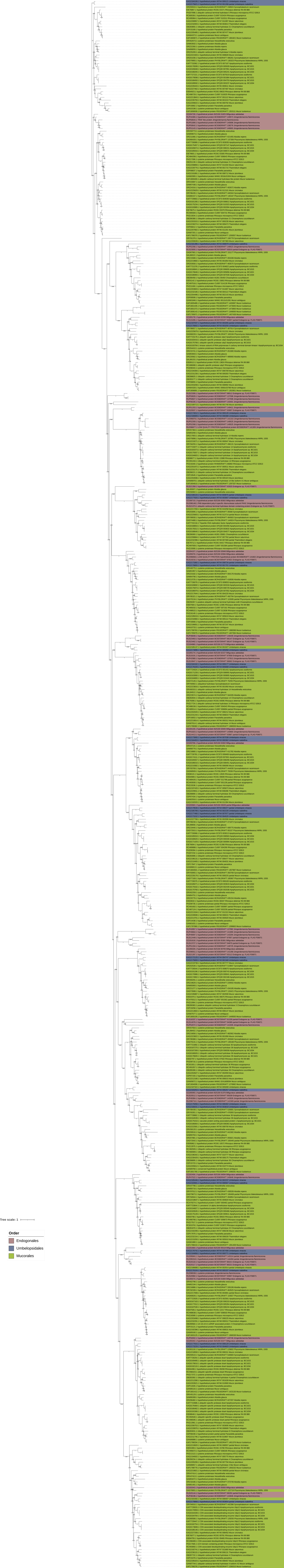
Tree scale: 1
Order
Endogonales
Umbelopsidales
Mucorales
