# Supplementary material for: Low-complexity regions in fungi display functional groups and are depleted in positively charged amino acids
Source: NAR Genom Bioinform. 2025 Feb 27;7(1):lqaf014. doi: 10.1093/nargab/lqaf014 (PMC11878562; doi:10.1093/nargab/lqaf014)
Supplement: lqaf014_Supplemental_Files [file lqaf014_supplemental_files.zip › S2.zip!/S2/AK_LCR_jnb_2.html]

lcr\_supplement\_2


In [1]:

```
# Import libraries
import os
from io import StringIO
import matplotlib.pyplot as plt
from matplotlib.patches import Patch
import numpy as np
import pandas as pd
import seaborn as sns
from collections import Counter
from scipy import stats
from scipy.stats import mannwhitneyu
```

In [2]:

```
## Specifying mappings of proteome IDs to their respective phylum
```

In [3]:

```
gcatax_data = """
GCA_000002525.1	Ascomycota
GCA_000146045.2	Ascomycota
GCA_000146945.2	Ascomycota
GCA_000149335.2	Ascomycota
GCA_000149845.2	Ascomycota
GCA_000150675.2	Ascomycota
GCA_000151645.1	Ascomycota
GCA_000167675.2	Ascomycota
GCA_000182965.3	Ascomycota
GCA_000219625.1	Ascomycota
GCA_000225545.1	Ascomycota
GCA_000230625.1	Ascomycota
GCA_001477545.1	Ascomycota
GCA_001929475.1	Ascomycota
GCA_012184305.1	Ascomycota
GCA_012184315.1	Ascomycota
GCA_905067625.1	Ascomycota
GCA_019456465.1	Ascomycota
GCA_000002945.2	Ascomycota
GCA_000182925.2	Ascomycota
GCA_000149205.2	Ascomycota
GCA_000006255.1	Basidiomycota
GCA_000181695.1	Basidiomycota
GCA_000182895.1	Basidiomycota
GCA_000204055.1	Basidiomycota
GCA_000218685.1	Basidiomycota
GCA_000328475.2	Basidiomycota
GCA_000400465.1	Basidiomycota
GCA_000711695.1	Basidiomycota
GCA_000827485.1	Basidiomycota
GCA_015039355.1	Basidiomycota
GCA_016906535.1	Basidiomycota
GCA_000149245.3	Basidiomycota
GCA_000149925.1	Basidiomycota
GCA_000708205.1	Basidiomycota
GCA_000292625.1	Basidiomycota
GCA_000151295.1	Blastocladiomycota
GCA_002102555.1	Blastocladiomycota
GCA_000182565.2	Chytridiomycota
GCA_000203795.1	Chytridiomycota
GCA_001574975.1	Chytridiomycota
GCA_002104985.1	Chytridiomycota
GCA_003614705.1	Chytridiomycota
GCA_003615045.1	Chytridiomycota
GCA_006535955.1	Chytridiomycota
GCA_006535965.1	Chytridiomycota
GCA_006535975.1	Chytridiomycota
GCA_006535985.1	Chytridiomycota
GCA_006536005.1	Chytridiomycota
GCA_000439145.3	Glomeromycotina
GCA_003547095.1	Glomeromycotina
GCA_003549995.1	Glomeromycotina
GCA_003550305.1	Glomeromycotina
GCA_003550325.1	Glomeromycotina
GCA_009809945.1	Glomeromycotina
GCA_015698045.1	Glomeromycotina
GCA_016748015.1	Glomeromycotina
GCA_001953115.1	Kickxellomycotina
GCA_001969505.1	Kickxellomycotina
GCA_001970855.1	Kickxellomycotina
GCA_002104995.1	Kickxellomycotina
GCA_002705745.1	Kickxellomycotina
GCA_003086715.1	Kickxellomycotina
GCA_003086725.1	Kickxellomycotina
GCA_003086735.1	Kickxellomycotina
GCA_003097675.1	Kickxellomycotina
GCA_003614675.1	Kickxellomycotina
GCA_000146465.1	Microsporidia
GCA_000192795.1	Microsporidia
GCA_000209485.1	Microsporidia
GCA_000230595.3	Microsporidia
GCA_000231115.1	Microsporidia
GCA_000250985.1	Microsporidia
GCA_000277815.3	Microsporidia
GCA_000280035.2	Microsporidia
GCA_000316135.1	Microsporidia
GCA_000383075.1	Microsporidia
GCA_000385875.2	Microsporidia
GCA_000447185.1	Microsporidia
GCA_000738915.1	Microsporidia
GCA_000760515.2	Microsporidia
GCA_000803265.1	Microsporidia
GCA_000988165.1	Microsporidia
GCA_001432165.1	Microsporidia
GCA_001642395.1	Microsporidia
GCA_001642415.1	Microsporidia
GCA_001875675.1	Microsporidia
GCA_002081675.1	Microsporidia
GCA_002087885.1	Microsporidia
GCA_002087915.1	Microsporidia
GCA_004000155.1	Microsporidia
GCA_004325065.1	Microsporidia
GCA_004325075.1	Microsporidia
GCA_014805555.1	Microsporidia
GCA_014805705.1	Microsporidia
GCA_015832245.1	Microsporidia
GCA_016255985.1	Microsporidia
GCA_016256075.1	Microsporidia
GCA_000739165.1	Mortierellomycotina
GCA_001651415.1	Mortierellomycotina
GCA_002105155.1	Mortierellomycotina
GCA_015499975.1	Mortierellomycotina
GCA_015499985.1	Mortierellomycotina
GCA_015500005.1	Mortierellomycotina
GCA_015500155.1	Mortierellomycotina
GCA_015502445.1	Mortierellomycotina
GCA_015502455.1	Mortierellomycotina
GCA_015502465.1	Mortierellomycotina
GCA_015502475.1	Mortierellomycotina
GCA_015502485.1	Mortierellomycotina
GCA_015502505.1	Mortierellomycotina
GCA_015502575.1	Mortierellomycotina
GCA_015529505.1	Mortierellomycotina
GCA_015529515.1	Mortierellomycotina
GCA_015529795.1	Mortierellomycotina
GCA_015679515.1	Mortierellomycotina
GCA_015679535.1	Mortierellomycotina
GCA_015679545.1	Mortierellomycotina
GCA_015679585.1	Mortierellomycotina
GCA_015679605.1	Mortierellomycotina
GCA_015679695.1	Mortierellomycotina
GCA_015680625.1	Mortierellomycotina
GCA_015680635.1	Mortierellomycotina
GCA_015847235.1	Mortierellomycotina
GCA_015849295.1	Mortierellomycotina
GCA_015849325.1	Mortierellomycotina
GCA_015849335.1	Mortierellomycotina
GCA_015852385.1	Mortierellomycotina
GCA_015852425.1	Mortierellomycotina
GCA_015852495.1	Mortierellomycotina
GCA_016097855.1	Mortierellomycotina
GCA_016097895.1	Mortierellomycotina
GCA_016097935.1	Mortierellomycotina
GCA_016097945.1	Mortierellomycotina
GCA_016098005.1	Mortierellomycotina
GCA_016098015.1	Mortierellomycotina
GCA_016098025.1	Mortierellomycotina
GCA_016098035.1	Mortierellomycotina
GCA_016098045.1	Mortierellomycotina
GCA_016098085.1	Mortierellomycotina
GCA_016098105.1	Mortierellomycotina
GCA_016098155.1	Mortierellomycotina
GCA_016098165.1	Mortierellomycotina
GCA_019671135.1	Mortierellomycotina
GCA_000149305.1	Mucoromycotina
GCA_000938895.1	Mucoromycotina
GCA_000950595.1	Mucoromycotina
GCA_001638985.2	Mucoromycotina
GCA_001683725.1	Mucoromycotina
GCA_002104935.1	Mucoromycotina
GCA_002105135.1	Mucoromycotina
GCA_002105175.1	Mucoromycotina
GCA_002261195.1	Mucoromycotina
GCA_002708625.1	Mucoromycotina
GCA_003325435.1	Mucoromycotina
GCA_003990745.1	Mucoromycotina
GCA_003990785.1	Mucoromycotina
GCA_010203745.1	Mucoromycotina
GCA_014839865.1	Mucoromycotina
GCA_016097715.1	Mucoromycotina
GCA_016097735.1	Mucoromycotina
GCA_016097755.1	Mucoromycotina
GCA_016758895.1	Mucoromycotina
GCA_016758905.1	Mucoromycotina
GCA_016758935.1	Mucoromycotina
GCA_016758945.1	Mucoromycotina
GCA_016758965.1	Mucoromycotina
GCA_016758985.1	Mucoromycotina
GCA_900079185.1	Mucoromycotina
GCA_002104895.1	Neocallimastigomycotina
GCA_002104945.1	Neocallimastigomycotina
GCA_002104975.1	Neocallimastigomycotina
GCA_002157105.1	Neocallimastigomycotina
GCA_016946835.1	Neocallimastigomycotina
GCA_017911155.1	Olpidiomycota
GCA_000442015.1	Rozellomycota
GCA_002794465.1	Rozellomycota
GCA_001566745.1	Zoopagomycota
GCA_002104905.1	Zoopagomycota
GCA_003614145.1	Zoopagomycota
GCA_003614735.1	Zoopagomycota
GCA_003614755.1	Zoopagomycota
GCA_014839935.1	Zoopagomycota
"""
```

In [65]:

```
# Converting 'gcatax_data' into dictionary
gcatax_dict = {}
for line in gcatax_data.strip().split("\n"):
    gca, phylum = line.split("\t")
    gcatax_dict[gca] = phylum
    
# Coverting 'gcatax_data' into the dataframe
    
df_gcatax = pd.DataFrame(list(gcatax_dict.items()), columns=["Proteome ID", "Phylum"])

#df_gcatax.head()
```

In [5]:

```
# Define phylum colors for visualization
phylum_colors = {
    "Ascomycota": "#5e243d",
    "Basidiomycota": "#f8d846",
    "Chytridiomycota": "#44b0d7",
    "Glomeromycotina": "#9a77c0",
    "Kickxellomycotina": "#62a073",
    "Mortierellomycotina": "#5e87f1",
    "Mucoromycotina": "#c8b2e4",
    "Neocallimastigomycotina": "#cb5751",
    "Blastocladiomycota": "#e37e2d",
    "Microsporidia": "#a5b266",
    "Rozellomycota": "#b6b6af",
    "Olpidiomycota": "#6a6a6a",
    "Zoopagomycota": "#e4659a"
}
```

In [6]:

```
## Adjusting and preprocessing 'Main_Dataset.tsv' for further analysis
```

In [7]:

```
## Applying column names to the base dataset
```

In [8]:

```
base_filename = "Main_Dataset.tsv"

column_headers = [
    "Proteome ID", "Protein ID", "Prot Length", "Has Domain?", 
    "Domains Length Normalized", "Num Transmembranes", "Total Length TMs", 
    "TMs Normalized", "Has Signal Peptide?", "Num LCRs", "Total Length LCRs", 
    "LCRs Symbolic Categories", "LCRs Length Normalized", "LCR N-term", 
    "LCR Middle", "LCR C-term", "Pfam LCR", "Pfam Protein", "GO Terms"
]

base_df = pd.read_csv(base_filename, sep="\t", header=None, names=column_headers)

base_df = base_df.merge(df_gcatax, on='Proteome ID', how='left')
```

In [9]:

```
## Removing 'xxxxx' stretches from LCR seqeunces
```

In [10]:

```
# Filter base_df to exclude rows containing 'xxxxx' in specific columns
main_df = base_df[
    ~(
        base_df['LCR N-term'].str.contains('xxxxx', na=False) |
        base_df['LCR Middle'].str.contains('xxxxx', na=False) |
        base_df['LCR C-term'].str.contains('xxxxx', na=False)
    )
].copy()

# Calculate and print the length of the new DataFrame
main_df_length = len(main_df)
print(f"The number of rows in the filtered DataFrame (main_df): {main_df_length}")

# Display the first few rows of the new DataFrame
main_df.head()
```

```
The number of rows in the filtered DataFrame (main_df): 1882840
```

Out[10]:

|  | Proteome ID | Protein ID | Prot Length | Has Domain? | Domains Length Normalized | Num Transmembranes | Total Length TMs | TMs Normalized | Has Signal Peptide? | Num LCRs | Total Length LCRs | LCRs Symbolic Categories | LCRs Length Normalized | LCR N-term | LCR Middle | LCR C-term | Pfam LCR | Pfam Protein | GO Terms | Phylum |
| --- | --- | --- | --- | --- | --- | --- | --- | --- | --- | --- | --- | --- | --- | --- | --- | --- | --- | --- | --- | --- |
| 0 | GCA\_000182895.1 | EFI27490.1 | 1121 | 1 | ooo....oo. | 0 | 0 | .......... | 0 | 1 | 54 | ..o | .........o | NaN | NaN | aekkrleeeakqkeeaekaaraeakkakaaaanaakkarraqraae... | NaN | PF00682.23 PF00226.35 | catalytic activity{0003824} | Basidiomycota |
| 1 | GCA\_000219625.1 | EGP88038.1 | 1104 | 1 | o..o...... | 0 | 0 | .......... | 0 | 0 | 0 | ... | .......... | NaN | NaN | NaN | NaN | PF03810.23 PF13513.10 | small GTPase binding{0031267}\|intracellular pr... | Ascomycota |
| 2 | GCA\_003614675.1 | RKP38109.1 | 115 | 1 | ooooooooo. | 0 | 0 | .......... | 0 | 0 | 0 | ... | .......... | NaN | NaN | NaN | NaN | PF00075.28 | nucleic acid binding{0003676}\|RNA-DNA hybrid r... | Kickxellomycotina |
| 3 | GCA\_015679535.1 | KAF9321969.1 | 1044 | 1 | .oooo...oo | 0 | 0 | .......... | 0 | 0 | 0 | ... | .......... | NaN | NaN | NaN | NaN | PF13365.10 PF12812.11 PF12812.11 | NaN | Mortierellomycotina |
| 4 | GCA\_016098045.1 | KAG0234905.1 | 80 | 0 | .......... | 0 | 0 | .......... | 0 | 0 | 0 | ... | .......... | NaN | NaN | NaN | NaN | NaN | NaN | Mortierellomycotina |

In [11]:

```
## Splitting the main dataset into different cathegories
```

In [12]:

```
### All LCR containing proteins
```

In [13]:

```
df_alllcrs = main_df[main_df['Num LCRs'] > 0]
```

In [14]:

```
### LCRs overlapping with a protein domain
```

In [15]:

```
df_allindomain = main_df[(main_df['Pfam LCR'].notna()) & (main_df['Num LCRs'] > 0)]
```

In [16]:

```
### LCRs not overlapping with a protein domain
```

In [17]:

```
df_alloutdomain = main_df[(main_df['Pfam LCR'].isna()) & (main_df['Num LCRs'] > 0) & (main_df['Has Domain?'] == 1)]
```

In [18]:

```
### LCRs without a protein domain
```

In [19]:

```
df_allnodomain = main_df[(main_df['Has Domain?'] == 0) & (main_df['Num LCRs'] > 0)]
```

In [20]:

```
### Defining homopolymer LCRs
```

In [21]:

```
def has_homorepeat(sequence, repeat_length=5, percentage=0.7):
    # Skip sequences that are 'NULL' or empty
    if sequence == 'NULL' or pd.isnull(sequence):
        return False
    for i in range(len(sequence) - repeat_length + 1):
        if len(set(sequence[i:i+repeat_length])) == 1:
            repeat_char = sequence[i]
            if sequence.count(repeat_char) / len(sequence) >= percentage:
                return True
    return False

# Accesing columns
columns_to_check = [14, 15, 16] 

df_alllcrs_copy = df_alllcrs.copy()
df_alllcrs_copy = df_alllcrs_copy.astype(str)

# Identify proteins with homopolymers using numeric indices
homopolymers_indices = df_alllcrs_copy.apply(
    lambda row: any(has_homorepeat(row.iloc[col]) for col in columns_to_check if row.iloc[col] != 'NULL'),
    axis=1
)

# Filter rows with homopolymers
df_homoall = df_alllcrs_copy[homopolymers_indices].copy()
```

In [22]:

```
## Defining non-domain homopolymers
```

In [23]:

```
# Ensure columns are numeric where necessary
df_homoall['Has Domain?'] = pd.to_numeric(df_homoall['Has Domain?'], errors='coerce')
df_homoall['Num LCRs'] = pd.to_numeric(df_homoall['Num LCRs'], errors='coerce')

# Filter the DataFrame
df_homonodomain = df_homoall[(df_homoall['Has Domain?'] == 0) & (df_homoall['Num LCRs'] > 0)]
```

In [24]:

```
## Defining homopolymers overlaping and not overlaping with protein domain
```

In [25]:

```
df_homoall['Pfam LCR'] = df_homoall['Pfam LCR'].replace(['', 'None', 'nan'], np.nan)

df_homoall['Has Domain?'] = pd.to_numeric(df_homoall['Has Domain?'], errors='coerce').fillna(0).astype(int)
df_homoall['Num LCRs'] = pd.to_numeric(df_homoall['Num LCRs'], errors='coerce').fillna(0).astype(int)

# Filter for In Domain and Out Domain categories
df_homoindomain = df_homoall[
    df_homoall['Pfam LCR'].notna() &
    (df_homoall['Num LCRs'] > 0)
]

df_homooutdomain = df_homoall[
    df_homoall['Pfam LCR'].isna() &
    (df_homoall['Num LCRs'] > 0) &
    (df_homoall['Has Domain?'] == 1)
]

# Print lengths of the resulting DataFrames
print(f"Length of df_homoindomain: {len(df_homoindomain)}")
print(f"Length of df_homooutdomain: {len(df_homooutdomain)}")
```

```
Length of df_homoindomain: 2005
Length of df_homooutdomain: 15590
```

In [26]:

```
## Data for Figure 1A and Supplementary Figure S1
```

In [27]:

```
# Display the total number of proteins
dataset_size = len(main_df)
print(f"{dataset_size} number of all proteins")


# Initialize counters
count_dm_lcr = 0
count_dm_nolcr = 0
count_nodm_lcr = 0
count_nodm_nolcr = 0

# Iterate through each row to compute counts
for _, row in main_df.iterrows():
    has_domain = int(row['Has Domain?'])
    num_lcrs = int(row['Num LCRs'])
    
    if has_domain > 0 and num_lcrs > 0:  # Domain and LCR
        count_dm_lcr += 1
    elif has_domain > 0 and num_lcrs == 0:  # Domain but no LCR
        count_dm_nolcr += 1
    elif has_domain == 0 and num_lcrs > 0:  # No domain but LCR
        count_nodm_lcr += 1
    elif has_domain == 0 and num_lcrs == 0:  # Neither domain nor LCR
        count_nodm_nolcr += 1

# Print the counts
print(f"{count_dm_lcr} proteins with a domain and LCR")
print(f"{count_dm_nolcr} proteins with a domain but no LCR")
print(f"{count_nodm_lcr} proteins with no domain but LCR")
print(f"{count_nodm_nolcr} proteins with neither domain nor LCR")

# Compute percentages
perc_dm_lcr = round((count_dm_lcr / dataset_size) * 100, 2)
perc_dm_nolcr = round((count_dm_nolcr / dataset_size) * 100, 2)
perc_nodm_lcr = round((count_nodm_lcr / dataset_size) * 100, 2)
perc_nodm_nolcr = round((count_nodm_nolcr / dataset_size) * 100, 2)

# Print percentages
print(f"{perc_dm_lcr}% of all proteins have both a domain and an LCR")
print(f"{perc_dm_nolcr}% of all proteins have a domain but no LCR")
print(f"{perc_nodm_lcr}% of all proteins have no domain but an LCR")
print(f"{perc_nodm_nolcr}% of all proteins have neither a domain nor an LCR")
```

```
1882840 number of all proteins
253846 proteins with a domain and LCR
912405 proteins with a domain but no LCR
147914 proteins with no domain but LCR
568675 proteins with neither domain nor LCR
13.48% of all proteins have both a domain and an LCR
48.46% of all proteins have a domain but no LCR
7.86% of all proteins have no domain but an LCR
30.2% of all proteins have neither a domain nor an LCR
```

In [28]:

```
# Ensure correct data types
main_df['Prot Length'] = main_df['Prot Length'].astype(int)
main_df['Num Transmembranes'] = main_df['Num Transmembranes'].astype(int)
main_df['Num LCRs'] = main_df['Num LCRs'].astype(int)
main_df['Total Length LCRs'] = main_df['Total Length LCRs'].astype(int)
main_df['Total Length TMs'] = main_df['Total Length TMs'].astype(int)


# Index and value of protein with the maximum number of LCRs
i = main_df['Num LCRs'].idxmax()
max_nr_lcr = main_df.loc[i, 'Num LCRs']
print(f"Index of protein with max LCRs: {i}")
print(f"Max number of LCRs: {max_nr_lcr}")

# Sum of protein lengths and LCR lengths
sum_protein = main_df['Prot Length'].sum()
sum_lcr = main_df['Total Length LCRs'].sum()
coverage = round((sum_lcr / sum_protein) * 100, 2)
print(f"{sum_protein} total protein length, {sum_lcr} total LCR length, {coverage}% coverage by LCRs")

# Counts for different categories
count_dm = main_df['Has Domain?'].sum()
count_nd = dataset_size - count_dm
perc_dm = round((count_dm / dataset_size) * 100, 2)
perc_nd = round((count_nd / dataset_size) * 100, 2)
print(f"{count_dm} proteins with a domain, {perc_dm}% of all proteins")
print(f"{count_nd} proteins with no domain, {perc_nd}% of all proteins")

# Proteins with and without LCRs
count_lcr = (main_df['Num LCRs'] > 0).sum()
count_nlcr = dataset_size - count_lcr
perc_lcr = round((count_lcr / dataset_size) * 100, 2)
perc_nlcr = round((count_nlcr / dataset_size) * 100, 2)
print(f"{count_lcr} proteins with LCRs, {perc_lcr}% of all proteins")
print(f"{count_nlcr} proteins with no LCRs, {perc_nlcr}% of all proteins")

# Proteins with and without signal peptides
count_sign = (main_df['Has Signal Peptide?'] == 1).sum()
count_nosign = dataset_size - count_sign
perc_sign = round((count_sign / dataset_size) * 100, 2)
perc_nosign = round((count_nosign / dataset_size) * 100, 2)
print(f"{count_sign} proteins with a signal peptide, {perc_sign}% of all proteins")
print(f"{count_nosign} proteins with no signal peptide, {perc_nosign}% of all proteins")

# Proteins with transmembrane regions
count_TM = (main_df['Num Transmembranes'] > 0).sum()
count_noTM = dataset_size - count_TM
perc_TM = round((count_TM / dataset_size) * 100, 2)
perc_noTM = round((count_noTM / dataset_size) * 100, 2)
print(f"{count_TM} proteins with TM regions, {perc_TM}% of all proteins")
print(f"{count_noTM} proteins with no TM regions, {perc_noTM}% of all proteins")

# Mann-Whitney U Test between LCR + domain and LCR without domain
prot_with_lcr_and_domain = main_df[(main_df['Has Domain?'] == 1) & (main_df['Num LCRs'] > 0)]['Prot Length']
prot_with_lcr_no_domain = main_df[(main_df['Has Domain?'] == 0) & (main_df['Num LCRs'] > 0)]['Prot Length']

stat, pval = mannwhitneyu(prot_with_lcr_and_domain, prot_with_lcr_no_domain, alternative='two-sided')
print(f"p={pval:.2e} Mann-Whitney U test for proteins with domain and without to have LCRs")

# Secreted proteins with LCRs
secreted_lcrs = main_df[(main_df['Num LCRs'] > 0) & (main_df['Has Signal Peptide?'] == 1)].shape[0]
perc_secreted_lcrs = round((secreted_lcrs / dataset_size) * 100, 2)
perc_of_all_secreted = round((secreted_lcrs / count_sign) * 100, 2) if count_sign > 0 else 0
print(f"{secreted_lcrs} secreted proteins with LCRs, {perc_secreted_lcrs}% of all proteins")
print(f"{perc_of_all_secreted}% of secreted proteins have LCRs")

# TM and LCR intersection analysis
TM_lcrs = main_df[(main_df['Num Transmembranes'] > 0) & (main_df['Num LCRs'] > 0)].shape[0]
perc_TM_lcrs = round((TM_lcrs / dataset_size) * 100, 2)
perc_of_all_TM = round((TM_lcrs / count_TM) * 100, 2) if count_TM > 0 else 0
print(f"{TM_lcrs} proteins with TM regions and LCRs")
print(f"{perc_TM_lcrs}% of all proteins have both TM regions and LCRs")
print(f"{perc_of_all_TM}% of proteins with TM regions also have LCRs")
```

```
Index of protein with max LCRs: 218630
Max number of LCRs: 125
822973490 total protein length, 20473059 total LCR length, 2.49% coverage by LCRs
1166251 proteins with a domain, 61.94% of all proteins
716589 proteins with no domain, 38.06% of all proteins
401760 proteins with LCRs, 21.34% of all proteins
1481080 proteins with no LCRs, 78.66% of all proteins
116079 proteins with a signal peptide, 6.17% of all proteins
1766761 proteins with no signal peptide, 93.83% of all proteins
313326 proteins with TM regions, 16.64% of all proteins
1569514 proteins with no TM regions, 83.36% of all proteins
p=0.00e+00 Mann-Whitney U test for proteins with domain and without to have LCRs
29746 secreted proteins with LCRs, 1.58% of all proteins
25.63% of secreted proteins have LCRs
64488 proteins with TM regions and LCRs
3.43% of all proteins have both TM regions and LCRs
20.58% of proteins with TM regions also have LCRs
```

In [29]:

```
## Figure 1B
```

In [30]:

```
# calculate the frequency of each LCR count
lcr_counts = df_alllcrs['Num LCRs'].value_counts().sort_index()

# create the plot
plt.figure(figsize=(14, 8))
plt.bar(lcr_counts.index, lcr_counts.values, color='darkgray')
plt.xlabel('Number of Low Complexity Regions (LCRs) per Protein')
plt.ylabel('Protein Count')
plt.title('Distribution of Low Complexity Regions in Proteins')
plt.xticks(rotation=0)

# set the y-axis to logarithmic scale with custom ticks
plt.yscale('log')
plt.yticks([1, 10, 1000, 100000, 1000000]) 

# display the plot
plt.tight_layout()
plt.show()
```

In [31]:

```
## FIgure 2A
```

In [32]:

```
# Count the number of proteins per proteome in each filtered dataframe
counts_in_domain = df_allindomain.groupby('Proteome ID').size().rename('In Domain LCR Count')
counts_out_domain = df_alloutdomain.groupby('Proteome ID').size().rename('Out Domain LCR Count')
counts_no_domain = df_allnodomain.groupby('Proteome ID').size().rename('No Domain LCR Count')

# Merge counts into the main dataframe
df_counts = pd.DataFrame({
    'Proteome ID': main_df['Proteome ID']
}).drop_duplicates()

df_counts = df_counts.merge(counts_in_domain, on='Proteome ID', how='left').fillna(0)
df_counts = df_counts.merge(counts_out_domain, on='Proteome ID', how='left').fillna(0)
df_counts = df_counts.merge(counts_no_domain, on='Proteome ID', how='left').fillna(0)

# Ensure counts are integers
df_counts[['In Domain LCR Count', 'Out Domain LCR Count', 'No Domain LCR Count']] = df_counts[
    ['In Domain LCR Count', 'Out Domain LCR Count', 'No Domain LCR Count']
].astype(int)

# Merge phylum information
df_counts = df_counts.merge(df_gcatax, on='Proteome ID', how='left')

# Calculate total LCR count
df_counts['Total LCR Count'] = (
    df_counts['In Domain LCR Count'] +
    df_counts['Out Domain LCR Count'] +
    df_counts['No Domain LCR Count']
)

# Merge the total proteome count from the main dataframe
df_counts = df_counts.merge(
    main_df[['Proteome ID', 'Protein ID']].groupby('Proteome ID').size().rename('Total Proteome Count').reset_index(),
    on='Proteome ID',
    how='left'
)

# Plot correlation between Total LCR Count and Total Proteome Count
def plot_correlations(df, x, y, title):
    fig, ax = plt.subplots(figsize=(10, 8))
    for phylum, color in phylum_colors.items():
        subset = df[df['Phylum'] == phylum]
        if not subset.empty:
            ax.scatter(subset[x], subset[y], color=color, label=phylum)

    ax.set_xlabel(x)
    ax.set_ylabel(y)
    r, p = stats.pearsonr(df[x], df[y])
    ax.text(0.05, 0.95, f'r={r:.2f}, p={p:.2e}', transform=ax.transAxes)
    ax.legend(title='Phylum', bbox_to_anchor=(1.05, 1), loc='upper left', frameon=False)
    plt.tight_layout(rect=[0, 0, 0.85, 1])  # Adjust layout to make room for the legend
    plt.box(False)
    plt.title(title)
    plt.show()
    plt.close(fig)

plot_correlations(df_counts, 'Total Proteome Count', 'Total LCR Count', 'Correlation between Total LCR Count and Total Proteome Count')
```

In [33]:

```
## Figure 2B
```

In [34]:

```
# Calculate total LCR counts for each proteome from relevant dataframes
lcr_counts = (
    df_allindomain.groupby('Proteome ID').size().rename('In Domain LCR Count').reset_index()
    .merge(df_alloutdomain.groupby('Proteome ID').size().rename('Out Domain LCR Count').reset_index(), on='Proteome ID', how='outer')
    .merge(df_allnodomain.groupby('Proteome ID').size().rename('No Domain LCR Count').reset_index(), on='Proteome ID', how='outer')
    .fillna(0)
)

# Calculate Total LCR Count
lcr_counts['Total LCR Count'] = (
    lcr_counts['In Domain LCR Count'] +
    lcr_counts['Out Domain LCR Count'] +
    lcr_counts['No Domain LCR Count']
)

# Merge with phylum information
lcr_counts = lcr_counts.merge(df_gcatax, on='Proteome ID', how='left')

# Define the order of phyla
phylum_order = [
    "Microsporidia", "Rozellomycota", "Chytridiomycota", "Neocallimastigomycotina",
    "Blastocladiomycota", "Olpidiomycota", "Zoopagomycotina", "Kickxellomycotina",
    "Glomeromycotina", "Mortierellomycotina", "Mucoromycotina", "Basidiomycota",
    "Ascomycota"
]

# Plotting
plt.figure(figsize=(12, 8))
sns.boxplot(
    x='Total LCR Count', 
    y='Phylum', 
    data=lcr_counts, 
    order=phylum_order, 
    color='darkgrey', 
    linewidth=0.5
)

plt.xlabel('Total LCR Count')
plt.ylabel('Phylum')
plt.title('Total Abundance of Proteins with LCRs per Phylum')
plt.box(False) 
plt.tight_layout()
plt.show()
```

In [35]:

```
## Figure 3A
```

In [36]:

```
# Define known phyla for filtering
known_phyla = {
    "Ascomycota", "Basidiomycota", "Blastocladiomycota", "Chytridiomycota",
    "Glomeromycotina", "Kickxellomycotina", "Microsporidia", "Mortierellomycotina",
    "Mucoromycotina", "Neocallimastigomycotina", "Olpidiomycota", "Rozellomycota",
    "Zoopagomycota"
}

# Function to extract LCR positions from 'LCRs Length Normalized'
def extract_lcr_positions(df):
    df = df[df['Phylum'].isin(known_phyla)]
    df['LCR_Positions'] = df['LCRs Length Normalized'].apply(
        lambda x: [i + 1 for i, char in enumerate(str(x)) if char == 'o'] if pd.notna(x) else []
    )
    return df

# Apply extraction to the `df_alllcrs` dataframe
df_alllcrs_processed = extract_lcr_positions(df_alllcrs)

# Prepare data structure for accumulating LCR positions
max_position = df_alllcrs_processed['LCRs Length Normalized'].str.len().max()
total_counts = pd.Series(0, index=range(1, max_position + 1))

# Analyze LCR positions
for positions in df_alllcrs_processed['LCR_Positions']:
    for pos in positions:
        total_counts.at[pos] += 1

# Plot the overall frequency of homorepeat positions
plt.figure(figsize=(10, 4))
total_counts.plot(kind='bar', color='darkgray', width=0.75)
plt.title('General Frequency of All LCR Positions')
plt.xlabel('LCR Position')
plt.ylabel('Frequency')
plt.xticks(range(len(total_counts)), total_counts.index, rotation=0)
plt.box(False)
plt.tight_layout()
plt.show()
```

In [37]:

```
## FIgures 3B, C, D + Supplementary Figures S9A - J
```

In [38]:

```
# Define known phyla for analysis
known_phyla = {
    "Ascomycota", "Basidiomycota", "Blastocladiomycota", "Chytridiomycota",
    "Glomeromycotina", "Kickxellomycotina", "Microsporidia", "Mortierellomycotina",
    "Mucoromycotina", "Neocallimastigomycotina", "Olpidiomycota", "Rozellomycota",
    "Zoopagomycota"
}

# Convert the set of known phyla to a sorted list
known_phyla_list = sorted(list(known_phyla))

# Extract and process LCR positions from the dataframe
def process_lcr_positions(df):
    # Filter known phyla
    df = df[df['Phylum'].isin(known_phyla_list)]
    # Extract LCR positions from the 'LCRs Length Normalized' column
    df['LCR_Positions'] = df['LCRs Length Normalized'].apply(
        lambda x: [i + 1 for i, char in enumerate(str(x)) if char == 'o'] if pd.notna(x) else []
    )
    return df

# Process the existing dataframe
df_alllcrs_processed = process_lcr_positions(df_alllcrs)

# Prepare data structure for accumulating LCR positions
max_position = df_alllcrs_processed['LCRs Length Normalized'].str.len().max()
counts = pd.DataFrame(0, index=range(1, max_position + 1), columns=known_phyla_list)

# Analyze LCR positions by phylum
for phylum in known_phyla_list:
    phylum_data = df_alllcrs_processed[df_alllcrs_processed['Phylum'] == phylum]['LCR_Positions']
    for positions in phylum_data:
        for pos in positions:
            counts.at[pos, phylum] += 1

# Plot and show frequency of LCR positions for each phylum
for phylum in known_phyla_list:
    plt.figure(figsize=(10, 4))
    counts[phylum].plot(kind='bar', color='darkgray', width=0.75)
    plt.title(f'Frequency of LCR Positions for {phylum}')
    plt.xlabel('LCR Position')
    plt.ylabel('Frequency')
    plt.xticks(range(len(counts)), counts.index, rotation=0)
    plt.box(False)
    plt.show()
```

In [39]:

```
## Figure 4A
```

In [40]:

```
# Use the existing dataframe df_alllcrs
all_lcr_data = df_alllcrs.copy()

# Filter out rows where 'Total Length LCRs' is NULL
all_lcr_data = all_lcr_data[all_lcr_data['Total Length LCRs'] != 'NULL']

# Convert 'Total Length LCRs' column to numeric
all_lcr_data['Total Length LCRs'] = pd.to_numeric(all_lcr_data['Total Length LCRs'])


# Calculate total length of homorepeats for each row
def calculate_homopolymer_lengths(row):
    sequences = []
    for col in ['LCR N-term', 'LCR Middle', 'LCR C-term']:
        if pd.notnull(row[col]):
            sequences.extend(row[col].split(','))
    total_length = 0
    for seq in sequences:
        seq = seq.replace(',', '')  
        if has_homorepeat(seq.lower()):  # Convert to lowercase
            total_length += len(seq)
    return total_length

# Apply calculation for all LCRs dataset
all_lcr_data['Homopolymer Lengths'] = all_lcr_data.apply(calculate_homopolymer_lengths, axis=1)

# Plot histograms
plt.figure(figsize=(10, 8))
plt.hist(all_lcr_data['Total Length LCRs'], bins=np.arange(0, 125, 5), color='#A0A0A0', alpha=0.7, label='Complex LCRs')
plt.hist(all_lcr_data[all_lcr_data['Homopolymer Lengths'] > 0]['Homopolymer Lengths'], bins=np.arange(0, 125, 5), color='#606060', alpha=0.7, label='Homopolymers')

plt.xlabel("LCR sequence length")
plt.ylabel("Count of sequences")
plt.xticks(np.arange(0, 125, 10))
plt.legend(loc='upper right', frameon=False)
plt.box(False)
plt.tight_layout()
plt.show()
```

In [41]:

```
## Figure 4B
```

In [42]:

```
# Known phyla for analysis
known_phyla = {
    "Ascomycota", "Basidiomycota", "Blastocladiomycota", "Chytridiomycota",
    "Glomeromycotina", "Kickxellomycotina", "Microsporidia", "Mortierellomycotina",
    "Mucoromycotina", "Neocallimastigomycotina", "Olpidiomycota", "Rozellomycota",
    "Zoopagomycota"
}

# Ensure 'Phylum' column and filter for known phyla
df_homoall['Phylum'] = df_homoall['Phylum'].astype(str)
df_homoall = df_homoall[df_homoall['Phylum'].isin(known_phyla)]

# Function to extract LCR positions
def get_lcr_positions(symbolic_category):
    """
    Extracts positions of 'o' characters in the LCR symbolic categories.
    """
    if pd.isnull(symbolic_category):
        return []
    return [i + 1 for i, char in enumerate(symbolic_category) if char == 'o']

# Add LCR positions as a new column
df_homoall['LCR_Positions'] = df_homoall['LCRs Length Normalized'].apply(get_lcr_positions)

# Prepare data structure for accumulating LCR positions
total_counts = pd.Series(0, index=range(1, 11))

# Analyze LCR positions by phylum
for phylum in known_phyla:
    phylum_data = df_homoall[df_homoall['Phylum'] == phylum]['LCR_Positions']
    for positions in phylum_data:
        for pos in positions:
            if pos <= 10:  # Restrict to positions 1-10 for the bar chart
                total_counts.at[pos] += 1

# Plot the overall frequency of homorepeat positions across all phyla
plt.figure(figsize=(10, 4))
total_counts.plot(kind='bar', color='darkgray', width=0.75)
plt.title('General Frequency of Homorepeat Positions')
plt.xlabel('Homorepeat Position')
plt.ylabel('Frequency')
plt.xticks(range(0, 10), range(1, 11), rotation=0)
plt.box(False)
plt.tight_layout()
plt.show()
```

In [43]:

```
## Figure 5A
```

In [44]:

```
# Use the df_homoall dataframe directly
df = df_homoall

# Calculate the protein counts per proteome
proteins_per_proteome = df.groupby(['Proteome ID', 'Phylum']).size().reset_index(name='Protein Count')

# Define the phylum order
phylum_order = [
    'Microsporidia',
    'Rozellomycota',
    'Chytridiomycota',
    'Neocallimastigomycotina',
    'Blastocladiomycota',
    'Olpidiomycota',
    'Kickxellomycotina',
    'Zoopagomycota',
    'Glomeromycotina',
    'Mortierellomycotina',
    'Mucoromycotina',
    'Basidiomycota',
    'Ascomycota'
]

# Filter and reorder data
proteins_per_proteome['Phylum'] = pd.Categorical(proteins_per_proteome['Phylum'], categories=phylum_order[::-1], ordered=True)

# Create the boxplot
plt.figure(figsize=(10, 8))
sns.boxplot(
    data=proteins_per_proteome,
    y='Phylum',
    x='Protein Count',
    color='darkgrey',
    fliersize=3
)

# Format the plot
plt.gca().invert_yaxis() 
plt.xlabel('Protein Count', fontsize=12)
plt.ylabel('', fontsize=12)
plt.box(False) 
plt.tight_layout()

# Show the plot
plt.show()
```

In [45]:

```
# Figure 5B
```

In [46]:

```
def count_proteins_per_proteome(df, proteome_col):
    return df[proteome_col].value_counts()

# Count proteins in homo_all
homo_all_counts = count_proteins_per_proteome(df_homoall, 'Proteome ID')

# Count proteins in df_alllcrs
alllcrs_counts = count_proteins_per_proteome(df_alllcrs, 'Proteome ID')

# Combine unique Proteome IDs from both sources
all_proteomes = pd.Index(homo_all_counts.index).union(alllcrs_counts.index)

# Create a combined DataFrame with all Proteome IDs
combined_counts = pd.DataFrame({
    'Proteome ID': all_proteomes
})

# Add counts from both data sources, filling missing values with 0
combined_counts['homo_all_counts'] = combined_counts['Proteome ID'].map(homo_all_counts).fillna(0).astype(int)
combined_counts['alllcrs_counts'] = combined_counts['Proteome ID'].map(alllcrs_counts).fillna(0).astype(int)

# Display the resulting DataFrame
combined_counts.head(30)
```

Out[46]:

|  | Proteome ID | homo\_all\_counts | alllcrs\_counts |
| --- | --- | --- | --- |
| 0 | GCA\_000002525.1 | 57 | 1496 |
| 1 | GCA\_000002945.2 | 18 | 464 |
| 2 | GCA\_000006255.1 | 29 | 1284 |
| 3 | GCA\_000146045.2 | 79 | 1034 |
| 4 | GCA\_000146465.1 | 0 | 69 |
| 5 | GCA\_000146945.2 | 63 | 2115 |
| 6 | GCA\_000149205.2 | 58 | 1405 |
| 7 | GCA\_000149245.3 | 63 | 1906 |
| 8 | GCA\_000149305.1 | 219 | 2216 |
| 9 | GCA\_000149335.2 | 84 | 1353 |
| 10 | GCA\_000149845.2 | 33 | 638 |
| 11 | GCA\_000149925.1 | 193 | 2664 |
| 12 | GCA\_000150675.2 | 122 | 2217 |
| 13 | GCA\_000151295.1 | 322 | 7774 |
| 14 | GCA\_000151645.1 | 51 | 1491 |
| 15 | GCA\_000167675.2 | 154 | 2098 |
| 16 | GCA\_000181695.1 | 45 | 770 |
| 17 | GCA\_000182565.2 | 70 | 1549 |
| 18 | GCA\_000182895.1 | 91 | 2758 |
| 19 | GCA\_000182925.2 | 309 | 3655 |
| 20 | GCA\_000182965.3 | 194 | 1886 |
| 21 | GCA\_000192795.1 | 0 | 128 |
| 22 | GCA\_000203795.1 | 28 | 723 |
| 23 | GCA\_000204055.1 | 98 | 2175 |
| 24 | GCA\_000209485.1 | 6 | 148 |
| 25 | GCA\_000218685.1 | 35 | 1137 |
| 26 | GCA\_000219625.1 | 53 | 1615 |
| 27 | GCA\_000225545.1 | 138 | 2820 |
| 28 | GCA\_000230595.3 | 3 | 357 |
| 29 | GCA\_000230625.1 | 101 | 2003 |

In [47]:

```
combined_counts = combined_counts.rename(
    columns={'homo_all_counts': 'Homopolymer Count', 'alllcrs_counts': 'Complex LCR Count'}
)

# Add Phylum information from df_gcatax
data = combined_counts.merge(df_gcatax, left_on='Proteome ID', right_on='Proteome ID', how='left')
data.rename(columns={'Phylum': 'Phylum'}, inplace=True)

# Function to plot correlation
def plot_correlation(data, x, y, xlabel, ylabel, title):
    fig, ax = plt.subplots(figsize=(10, 8))
    
    # Define colors per phylum (ensure phylum_colors is defined somewhere in your code)
    for phylum, color in phylum_colors.items():
        subset = data[data['Phylum'] == phylum]
        ax.scatter(subset[x], subset[y], color=color, label=phylum, alpha=0.6)

    # Label axes and title
    ax.set_xlabel(xlabel)
    ax.set_ylabel(ylabel)
    ax.set_title(title)

    # Calculate Pearson correlation
    r, p = stats.pearsonr(data[x].dropna(), data[y].dropna())
    p_formatted = f"{p:.2f}" if p >= 0.01 else f"{p:.1e}"

    ax.text(0.05, 0.95, f'r={r:.2f}, p={p_formatted}', ha='left', va='center', transform=ax.transAxes)


    # Add legend
    legend = ax.legend(frameon=False, title='Phylum', bbox_to_anchor=(1.05, 1), loc='upper left')
    plt.tight_layout()

    # Show plot
    plt.box(False)
    plt.show()

# Plot the correlation
plot_correlation(
    data, 
    'Complex LCR Count', 
    'Homopolymer Count', 
    'Complex LCR Count', 
    'Homopolymer Count', 
    ''
)
```

In [48]:

```
# Figure 6
```

In [49]:

```
phylum_order = [
    "Microsporidia", "Rozellomycota", "Chytridiomycota", "Neocallimastigomycotina",
    "Blastocladiomycota", "Olpidiomycota", "Zoopagomycota", "Kickxellomycotina",
    "Glomeromycotina", "Mortierellomycotina", "Mucoromycotina", "Basidiomycota",
    "Ascomycota"
]

def categorize_data(df, has_domain_col, num_lcrs_col, within_domain_col):
    df[within_domain_col] = df[within_domain_col].replace(['', 'None', 'nan'], np.nan)
    df[has_domain_col] = pd.to_numeric(df[has_domain_col], errors='coerce').fillna(0).astype(int)
    df[num_lcrs_col] = pd.to_numeric(df[num_lcrs_col], errors='coerce').fillna(0).astype(int)
    
    conditions = [
        df[within_domain_col].notna(),
        (df[has_domain_col] == 0) & (df[num_lcrs_col] >= 1),
        (df[has_domain_col] == 1) & df[within_domain_col].isna()
    ]
    choices = ['In Domain', 'Non-Domain', 'Out Domain']
    df['Category'] = np.select(conditions, choices, default='Unclassified')
    return df

data_lcrs = categorize_data(df_alllcrs.copy(), 'Has Domain?', 'Num LCRs', 'Pfam LCR')
data_homos = categorize_data(df_homoall.copy(), 'Has Domain?', 'Num LCRs', 'Pfam LCR')

def calculate_adjusted_ratios(df):
    grouped = df.groupby(['Phylum', 'Category']).size().unstack(fill_value=0)
    grouped['Total'] = grouped.sum(axis=1)
    ratios = grouped.iloc[:, :-1].div(grouped['Total'], axis=0) * 100
    ratios_adjusted = ratios.round(2)
    return grouped, ratios_adjusted

grouped_lcrs, ratios_adjusted_lcrs = calculate_adjusted_ratios(data_lcrs)
grouped_homos, ratios_adjusted_homos = calculate_adjusted_ratios(data_homos)

combined_lcrs = pd.concat([ratios_adjusted_lcrs.add_suffix('_percentage')], axis=1, keys=['Complex LCRs'])
combined_homos = pd.concat([ratios_adjusted_homos.add_suffix('_percentage')], axis=1, keys=['Homopolymers'])

categories_combined_all = pd.concat([combined_lcrs, combined_homos], axis=1)
categories_combined_all = categories_combined_all.loc[phylum_order]

# Display the combined DataFrame
categories_combined_all.head(30)
```

Out[49]:

|  | Complex LCRs | | | Homopolymers | | |
| --- | --- | --- | --- | --- | --- | --- |
| Category | In Domain\_percentage | Non-Domain\_percentage | Out Domain\_percentage | In Domain\_percentage | Non-Domain\_percentage | Out Domain\_percentage |
| Phylum |  |  |  |  |  |  |
| Microsporidia | 7.62 | 62.34 | 30.04 | 6.42 | 64.22 | 29.36 |
| Rozellomycota | 9.92 | 33.03 | 57.05 | 10.53 | 47.37 | 42.11 |
| Chytridiomycota | 7.76 | 36.02 | 56.22 | 6.80 | 38.24 | 54.97 |
| Neocallimastigomycotina | 5.22 | 44.96 | 49.81 | 3.51 | 52.23 | 44.26 |
| Blastocladiomycota | 6.62 | 40.63 | 52.75 | 4.87 | 45.58 | 49.56 |
| Olpidiomycota | 3.38 | 58.68 | 37.94 | 5.45 | 64.24 | 30.30 |
| Zoopagomycota | 8.73 | 42.88 | 48.39 | 9.38 | 44.47 | 46.15 |
| Kickxellomycotina | 7.57 | 41.48 | 50.95 | 8.97 | 37.67 | 53.36 |
| Glomeromycotina | 4.84 | 52.26 | 42.90 | 6.38 | 47.81 | 45.82 |
| Mortierellomycotina | 6.23 | 34.26 | 59.51 | 7.14 | 38.33 | 54.53 |
| Mucoromycotina | 7.06 | 29.69 | 63.25 | 6.57 | 29.70 | 63.73 |
| Basidiomycota | 7.52 | 39.13 | 53.35 | 8.26 | 38.04 | 53.70 |
| Ascomycota | 9.44 | 32.49 | 58.08 | 11.64 | 35.36 | 53.01 |

In [50]:

```
# Define phylum order
phylum_order = [
    "Microsporidia", "Rozellomycota", "Chytridiomycota", "Neocallimastigomycotina",
    "Blastocladiomycota", "Olpidiomycota", "Zoopagomycota", "Kickxellomycotina",
    "Glomeromycotina", "Mortierellomycotina", "Mucoromycotina", "Basidiomycota",
    "Ascomycota"
]
phylum_order = phylum_order[::-1]  

# Define labels and colors
labels = ['In Domain', 'Non-Domain', 'Out Domain']
labels_percentage = ['In Domain_percentage', 'Non-Domain_percentage', 'Out Domain_percentage']
colors = ['#606060', '#A0A0A0', '#808080']

# Extract percentage data for Complex LCRs and Homopolymers
percentages_lcrs = categories_combined_all['Complex LCRs'][labels_percentage].copy()
percentages_homos = categories_combined_all['Homopolymers'][labels_percentage].copy()

# Ensure the phylum order is consistent
percentages_lcrs = percentages_lcrs.reindex(phylum_order)
percentages_homos = percentages_homos.reindex(phylum_order)

# Function to adjust percentages to sum to 100%
def adjust_percentages(percentages_df):
    for i, row in percentages_df.iterrows():
        diff = 100 - row.sum()
        while abs(diff) > 0.01:
            if diff > 0:
                row[row.idxmin()] += diff
            else:
                row[row.idxmax()] += diff
            diff = 100 - row.sum()
    return percentages_df.round(2)

# Adjust percentages for both datasets
percentages_adjusted_lcrs = adjust_percentages(percentages_lcrs)
percentages_adjusted_homos = adjust_percentages(percentages_homos)

# Plotting side-by-side with shared legend
fig, axs = plt.subplots(
    1, 2,
    figsize=(14, len(phylum_order) * 0.5),
    sharey=True
)

# Function to plot data
def plot_data(ax, percentages_adjusted, title):
    left = np.zeros(len(phylum_order))
    for idx, (label_perc, color) in enumerate(zip(labels_percentage, colors)):
        widths = percentages_adjusted[label_perc].values
        ax.barh(
            phylum_order,
            widths,
            left=left,
            color=color,
            label=labels[idx] if ax is axs[0] else None  # Only add legend labels to the first subplot
        )
        # Add percentage text labels on bars
        for i, (w, l) in enumerate(zip(widths, left)):
            if w > 0:
                ax.text(
                    l + w / 2,
                    i,
                    f"{w:.1f}",
                    ha='center',
                    va='center',
                    color='white',
                    fontsize=10
                )
        left += widths
    ax.set_title(title)
    ax.set_yticks(range(len(phylum_order)))  
    ax.set_yticklabels(phylum_order)       
    ax.invert_yaxis()  

# Plot data for Complex LCRs and Homopolymers
plot_data(axs[0], percentages_adjusted_lcrs, 'Complex LCRs (%)')
plot_data(axs[1], percentages_adjusted_homos, 'Homorepeats (%)')

# Style adjustments
for ax in axs:
    ax.set_facecolor('white')
    ax.spines['top'].set_visible(False)
    ax.spines['right'].set_visible(False)
    ax.spines['left'].set_visible(False)
    ax.spines['bottom'].set_visible(False)
    ax.tick_params(axis='x', which='both', bottom=False, labelbottom=False)
    ax.tick_params(axis='y', labelsize=12)

# Add legend outside the plots
legend_elements = [Patch(facecolor=color, label=label) for label, color in zip(labels, colors)]
fig.legend(
    handles=legend_elements,
    title='Category',
    bbox_to_anchor=(1, 1),
    loc='upper left',
    frameon=False,
    fontsize='large'
)

plt.tight_layout()
plt.show()
```

In [51]:

```
# Figure 7
```

In [52]:

```
def has_homorepeat(sequence, repeat_length=5, percentage=0.7):
    # Skip sequences that are 'NULL' or empty
    if sequence == 'NULL' or pd.isnull(sequence):
        return False
    for i in range(len(sequence) - repeat_length + 1):
        if len(set(sequence[i:i+repeat_length])) == 1:
            repeat_char = sequence[i]
            if sequence.count(repeat_char) / len(sequence) >= percentage:
                return True
    return False

columns_to_check = [14, 15, 16]

df_alllcrs_copy = df_alllcrs.copy()

df_alllcrs_copy = df_alllcrs_copy.astype(str)

homopolymers_indices = df_alllcrs_copy.apply(
    lambda row: any(has_homorepeat(row.iloc[col]) for col in columns_to_check if row.iloc[col] != 'NULL'),
    axis=1
)

# Filter rows with homopolymers
df_homoall = df_alllcrs_copy[homopolymers_indices].copy()
```

In [53]:

```
# General function to calculate amino acid frequencies
def calculate_aa_frequencies(df, sequence_columns, filter_function=None):
    aa_frequencies = Counter()
    total_aa_count = 0
    excluded_aa = {'X', 'B'}  # Exclude 'X' and 'B' from calculations

    # Iterate through each row in the dataframe
    for _, row in df.iterrows():
        # Collect sequences from specified columns
        sequences = []
        for col in sequence_columns:
            if pd.notnull(row[col]):  # Ensure the column is not null
                sequences_in_cell = row[col].split(',')
                sequences.extend(sequences_in_cell)  # Split sequences on commas if applicable

        # Apply filter function if provided
        if filter_function:
            sequences = [seq for seq in sequences if filter_function(seq)]

        # Count amino acids in sequences
        for seq in sequences:
            aa_frequencies.update(seq.upper())  # Convert to uppercase for consistency
            total_aa_count += len(seq)

    # Calculate frequencies as percentages (3 decimal places)
    if total_aa_count > 0:
        aa_frequencies = {
            aa: round((count / total_aa_count) * 100, 3)
            for aa, count in aa_frequencies.items()
            if aa.upper() not in excluded_aa  # Exclude 'X' and 'B'
        }
    else:
        aa_frequencies = {}

    return aa_frequencies

# Function to create a dataframe from frequencies
def frequencies_to_dataframe(frequencies_dict, column_name):
    df = pd.DataFrame.from_dict(frequencies_dict, orient='index', columns=[column_name])
    return df

# Initialize lists to collect dataframes
dataframes_list = []


# Split the dataframe into subsets based on 'Has Domain?'
df_homo_without_domain = df_homoall[df_homoall['Has Domain?'] == '0']
df_homo_with_domain = df_homoall[df_homoall['Has Domain?'] == '1']

# Calculate amino acid frequencies for each subset with homorepeat filter
sequence_columns = ['LCR N-term', 'LCR Middle', 'LCR C-term']
frequencies_homo_without_domain = calculate_aa_frequencies(
    df_homo_without_domain,
    sequence_columns,
    filter_function=has_homorepeat
)
frequencies_homo_with_domain = calculate_aa_frequencies(
    df_homo_with_domain,
    sequence_columns,
    filter_function=has_homorepeat
)

# Create dataframes for visualization
df_frequencies_homo_without_domain = frequencies_to_dataframe(frequencies_homo_without_domain, 'Homorepeats Without Domain')
df_frequencies_homo_with_domain = frequencies_to_dataframe(frequencies_homo_with_domain, 'Homorepeats With Domain')

# Merge the dataframes
df_homo_frequencies = df_frequencies_homo_without_domain.join(df_frequencies_homo_with_domain, how='outer').fillna(0)

# Collect the dataframe
dataframes_list.append(df_homo_frequencies)


# Ensure 'Has Domain?' is numeric
df_alllcrs = df_alllcrs.copy()
df_alllcrs['Has Domain?'] = df_alllcrs['Has Domain?'].astype(int)

# Split the dataframe into subsets based on 'Has Domain?'
df_lcrs_without_domain = df_alllcrs[df_alllcrs['Has Domain?'] == 0]
df_lcrs_with_domain = df_alllcrs[df_alllcrs['Has Domain?'] == 1]

# Calculate amino acid frequencies for each subset
frequencies_lcrs_without_domain = calculate_aa_frequencies(df_lcrs_without_domain, sequence_columns)
frequencies_lcrs_with_domain = calculate_aa_frequencies(df_lcrs_with_domain, sequence_columns)

# Create dataframes for visualization
df_frequencies_lcrs_without_domain = frequencies_to_dataframe(frequencies_lcrs_without_domain, 'Complex LCRs Without Domain')
df_frequencies_lcrs_with_domain = frequencies_to_dataframe(frequencies_lcrs_with_domain, 'Complex LCRs With Domain')

# Merge the dataframes
df_lcrs_frequencies = df_frequencies_lcrs_without_domain.join(df_frequencies_lcrs_with_domain, how='outer').fillna(0)

# Collect the dataframe
dataframes_list.append(df_lcrs_frequencies)

# Calculate amino acid frequencies for the entire dataframe
frequencies_lcrs_total = calculate_aa_frequencies(df_alllcrs, sequence_columns)

# Create a dataframe for visualization
df_frequencies_lcrs_total = frequencies_to_dataframe(frequencies_lcrs_total, 'All LCRs')

# Collect the dataframe
dataframes_list.append(df_frequencies_lcrs_total)


# Calculate frequencies for each region
frequencies_n_term = calculate_aa_frequencies(df_alllcrs, ['LCR N-term'])
frequencies_middle = calculate_aa_frequencies(df_alllcrs, ['LCR Middle'])
frequencies_c_term = calculate_aa_frequencies(df_alllcrs, ['LCR C-term'])

# Convert frequencies to dataframes
df_frequencies_n_term = frequencies_to_dataframe(frequencies_n_term, 'N-Terminus')
df_frequencies_middle = frequencies_to_dataframe(frequencies_middle, 'Middle')
df_frequencies_c_term = frequencies_to_dataframe(frequencies_c_term, 'C-Terminus')

# Merge the dataframes
df_lcrs_regions_frequencies = df_frequencies_n_term.join(
    [df_frequencies_middle, df_frequencies_c_term],
    how='outer'
).fillna(0)

# Collect the dataframe
dataframes_list.append(df_lcrs_regions_frequencies)

### Combine all dataframes into one

# Start with the first dataframe
combined_frequencies_df = dataframes_list[0]

# Merge the rest of the dataframes
for df in dataframes_list[1:]:
    combined_frequencies_df = combined_frequencies_df.join(df, how='outer').fillna(0)

# Sort the combined dataframe
combined_frequencies_df.sort_index(inplace=True)

# Display the combined dataframe
print("Combined Amino Acid Frequencies (%):")
combined_frequencies_df.head()
```

```
Combined Amino Acid Frequencies (%):
```

Out[53]:

|  | Homorepeats Without Domain | Homorepeats With Domain | Complex LCRs Without Domain | Complex LCRs With Domain | All LCRs | N-Terminus | Middle | C-Terminus |
| --- | --- | --- | --- | --- | --- | --- | --- | --- |
| A | 4.075 | 5.676 | 8.702 | 9.585 | 9.245 | 9.353 | 9.255 | 9.126 |
| C | 0.124 | 0.068 | 0.286 | 0.120 | 0.184 | 0.173 | 0.200 | 0.168 |
| D | 6.326 | 6.067 | 5.924 | 6.625 | 6.355 | 5.674 | 6.431 | 6.888 |
| E | 8.061 | 7.188 | 7.509 | 8.176 | 7.920 | 6.409 | 8.343 | 8.675 |
| F | 0.502 | 0.487 | 1.031 | 0.888 | 0.943 | 1.008 | 0.928 | 0.906 |

In [54]:

```
## Additional frequencies

### UniProt Background has been downloaded from UniProt website, Fungal Background calculated from all 183 proteomes used in this analysis
```

In [66]:

```
# Data for UniProt and Fungal backgrounds
data = {
    'A': [9, 7],
    'C': [1, 1],
    'D': [5, 6],
    'E': [6, 6],
    'F': [4, 4],
    'G': [7, 6],
    'H': [2, 2],
    'I': [6, 6],
    'K': [5, 6],
    'L': [10, 9],
    'M': [2, 2],
    'N': [4, 5],
    'P': [5, 5],
    'Q': [4, 4],
    'R': [6, 5],
    'S': [7, 9],
    'T': [6, 6],
    'V': [7, 6],
    'W': [1, 1],
    'Y': [3, 3],
}

# Column names for the DataFrame
columns = ['UniProt Background', 'Fungal Background']

# Create the DataFrame
background_df = pd.DataFrame.from_dict(data, orient='index', columns=columns)

# Display the DataFrame
#print("Background DataFrame:")
#background_df.head()
```

In [56]:

```
# Combine the dataframes in the specified order
def combine_dataframes(background_df, combined_frequencies_df):
    # Select and rename rows for the background data
    combined_data = pd.concat([
        background_df.rename(
            columns={"UniProt Background": "UniProt Background", 
                     "Fungal Background": "Fungal Background"}
        ).T,
        combined_frequencies_df.loc[:, [
            'All LCRs', 
            'Complex LCRs With Domain', 
            'Complex LCRs Without Domain', 
            'Homorepeats With Domain', 
            'Homorepeats Without Domain', 
            'N-Terminus', 
            'Middle', 
            'C-Terminus'
        ]].T.rename(
            index={
                'All LCRs': 'All LCRs',
                'Complex LCRs With Domain': 'Domain complex LCRs',
                'Complex LCRs Without Domain': 'Non-domain complex LCRs',
                'Homorepeats With Domain': 'Domain Homopolymers',
                'Homorepeats Without Domain': 'Non-domain Homopolymers',
                'N-Terminus': 'N-terminal',
                'Middle': 'middle',
                'C-Terminus': 'C-Terminus'
            }
        )
    ])

    # Set the categories (rows) as the index
    combined_data.index.name = 'Category'
    
    return combined_data

# Combine the dataframes
heatmap_data = combine_dataframes(background_df, combined_frequencies_df)

# Generate heatmap
def generate_heatmap(df, title, xlabel, ylabel, cmap="Greys", fmt=".2f"):
    # Generate heatmap
    plt.figure(figsize=(12, 8), dpi=300)
    sns.heatmap(
        df,
        cmap=cmap,
        annot=True,
        fmt=fmt,
        linewidths=0.5,
        vmax=df.values.max(),
        vmin=df.values.min(),
        annot_kws={'size': 10}
    )
    plt.title(title, fontsize=16)
    plt.xticks(rotation=45, ha='right', fontsize=10)  
    plt.yticks(rotation=0, fontsize=10)  
    plt.xlabel(xlabel, fontsize=12)
    plt.ylabel(ylabel, fontsize=12)
    plt.tight_layout()
    plt.show()

# Generate the heatmap
generate_heatmap(
    heatmap_data,
    title="Amino Acid Frequencies Heatmap",
    xlabel="Amino Acids",
    ylabel="Categories",
    cmap="Greys", 
    fmt=".0f"  
)
```

In [57]:

```
## Supplementary Figure S1
```

In [58]:

```
# Rename columns to shorter labels
label_mapping = {
    'Prot Length': 'len',
    'Has Domain?': 'dm',
    'Num Transmembranes': 'nr_tm',
    'Total Length TMs': 'len_tm',
    'Has Signal Peptide?': 'signal',
    'Num LCRs': 'nr_lcr',
    'Total Length LCRs': 'len_lcr'
}

correlation_matrix = main_df.rename(columns=label_mapping)[list(label_mapping.values())].corr()

# Custom function to format annotations
def format_values(val):
    if abs(val) < 0.01:  # Very small values in 4 decimals
        return f"{val:.4f}"
    return f"{val:.3f}"  # Round to 3 decimals otherwise

# Generate the heatmap
plt.figure(figsize=(8, 6))
sns.heatmap(
    correlation_matrix,
    annot=correlation_matrix.applymap(format_values),
    fmt="",  
    cmap="Blues",  
    square=True,  
    linewidths=0.5,  
    cbar_kws={"shrink": 0.8} 
)

# Add centered axis labels
plt.xticks(
    ticks=range(len(label_mapping)),
    labels=label_mapping.values(),
    rotation=45,
    ha='center',  
    fontsize=10
)
plt.yticks(
    ticks=range(len(label_mapping)),
    labels=label_mapping.values(),
    rotation=0,
    va='center',  
    fontsize=10
)

# Title (optional)
plt.title("Correlation Heatmap of Protein Attributes", fontsize=14)

# Show the plot
plt.tight_layout()
plt.show()
```

In [59]:

```
## Supplementary Figures S2, S3, S4, S8
```

In [60]:

```
def count_proteins_per_proteome(df, proteome_col):
    return df[proteome_col].value_counts()

# Count proteins in each dataframe
counts_homonodomain = count_proteins_per_proteome(df_homonodomain, 'Proteome ID')
counts_homooutdomain = count_proteins_per_proteome(df_homooutdomain, 'Proteome ID')
counts_homoindomain = count_proteins_per_proteome(df_homoindomain, 'Proteome ID')
counts_homoall = count_proteins_per_proteome(df_homoall, 'Proteome ID')
counts_main = count_proteins_per_proteome(main_df, 'Proteome ID')

# Combine all unique Proteome IDs
all_proteomes = (
    counts_homonodomain.index
    .union(counts_homooutdomain.index)
    .union(counts_homoindomain.index)
    .union(counts_homoall.index)
    .union(counts_main.index)
)

# Create a new consolidated DataFrame
consolidated_counts = pd.DataFrame({
    'Proteome ID': all_proteomes
})

# Add counts for each source, filling missing values with 0
consolidated_counts['Homo No Domain'] = consolidated_counts['Proteome ID'].map(counts_homonodomain).fillna(0).astype(int)
consolidated_counts['Homo Out Domain'] = consolidated_counts['Proteome ID'].map(counts_homooutdomain).fillna(0).astype(int)
consolidated_counts['Homo In Domain'] = consolidated_counts['Proteome ID'].map(counts_homoindomain).fillna(0).astype(int)
consolidated_counts['Homo All'] = consolidated_counts['Proteome ID'].map(counts_homoall).fillna(0).astype(int)
consolidated_counts['Main'] = consolidated_counts['Proteome ID'].map(counts_main).fillna(0).astype(int)

# Display the resulting DataFrame
consolidated_counts.reset_index(drop=True, inplace=True)

consolidated_counts.head(30)
```

Out[60]:

|  | Proteome ID | Homo No Domain | Homo Out Domain | Homo In Domain | Homo All | Main |
| --- | --- | --- | --- | --- | --- | --- |
| 0 | GCA\_000002525.1 | 22 | 31 | 4 | 57 | 6448 |
| 1 | GCA\_000002945.2 | 2 | 15 | 1 | 18 | 5132 |
| 2 | GCA\_000006255.1 | 13 | 16 | 0 | 29 | 9083 |
| 3 | GCA\_000146045.2 | 16 | 57 | 6 | 79 | 5997 |
| 4 | GCA\_000146465.1 | 0 | 0 | 0 | 0 | 1939 |
| 5 | GCA\_000146945.2 | 35 | 22 | 6 | 63 | 14491 |
| 6 | GCA\_000149205.2 | 13 | 39 | 6 | 58 | 9561 |
| 7 | GCA\_000149245.3 | 20 | 36 | 7 | 63 | 7826 |
| 8 | GCA\_000149305.1 | 88 | 115 | 16 | 219 | 17459 |
| 9 | GCA\_000149335.2 | 38 | 44 | 2 | 84 | 9910 |
| 10 | GCA\_000149845.2 | 7 | 24 | 2 | 33 | 4878 |
| 11 | GCA\_000149925.1 | 80 | 96 | 17 | 193 | 15979 |
| 12 | GCA\_000150675.2 | 38 | 73 | 11 | 122 | 10535 |
| 13 | GCA\_000151295.1 | 135 | 176 | 11 | 322 | 19447 |
| 14 | GCA\_000151645.1 | 18 | 29 | 4 | 51 | 7446 |
| 15 | GCA\_000167675.2 | 57 | 71 | 26 | 154 | 9115 |
| 16 | GCA\_000181695.1 | 9 | 28 | 8 | 45 | 4286 |
| 17 | GCA\_000182565.2 | 25 | 43 | 2 | 70 | 9422 |
| 18 | GCA\_000182895.1 | 46 | 42 | 3 | 91 | 13356 |
| 19 | GCA\_000182925.2 | 115 | 147 | 47 | 309 | 10813 |
| 20 | GCA\_000182965.3 | 41 | 106 | 47 | 194 | 6026 |
| 21 | GCA\_000192795.1 | 0 | 0 | 0 | 0 | 2773 |
| 22 | GCA\_000203795.1 | 7 | 18 | 3 | 28 | 8700 |
| 23 | GCA\_000204055.1 | 49 | 46 | 3 | 98 | 16372 |
| 24 | GCA\_000209485.1 | 2 | 4 | 0 | 6 | 3632 |
| 25 | GCA\_000218685.1 | 12 | 21 | 2 | 35 | 12925 |
| 26 | GCA\_000219625.1 | 23 | 25 | 5 | 53 | 10963 |
| 27 | GCA\_000225545.1 | 60 | 69 | 9 | 138 | 11479 |
| 28 | GCA\_000230595.3 | 3 | 0 | 0 | 3 | 4190 |
| 29 | GCA\_000230625.1 | 39 | 50 | 12 | 101 | 9578 |

In [61]:

```
# Merge proteome size information from df_gcatax
data = consolidated_counts.merge(df_gcatax, left_on='Proteome ID', right_on='Proteome ID', how='left')
data.rename(columns={'Phylum': 'Phylum', 'Main': 'Total Proteome Size'}, inplace=True)

# Function to plot correlation for each homopolymer category
def plot_correlation(data, x, y, xlabel, ylabel, title):
    fig, ax = plt.subplots(figsize=(10, 8))
    
    # Define colors per phylum (ensure phylum_colors is defined somewhere in your code)
    for phylum, color in phylum_colors.items():
        subset = data[data['Phylum'] == phylum]
        ax.scatter(subset[x], subset[y], color=color, label=phylum, alpha=0.6)

    # Label axes and title
    ax.set_xlabel(xlabel)
    ax.set_ylabel(ylabel)
    ax.set_title(title)

    # Calculate Pearson correlation
    r, p = stats.pearsonr(data[x].dropna(), data[y].dropna())
    ax.text(0.05, 0.95, f'r={r:.3f}, p={p:.3g}', ha='left', va='center', transform=ax.transAxes)

    # Add legend
    legend = ax.legend(frameon=False, title='Phylum', bbox_to_anchor=(1.05, 1), loc='upper left')
    plt.tight_layout()

    # Show plot
    plt.box(False)
    plt.show()

# List of homopolymer categories to plot
homopolymer_categories = [
    'Homo No Domain',
    'Homo Out Domain',
    'Homo In Domain',
    'Homo All'
]

# Generate plots for each homopolymer category
for category in homopolymer_categories:
    plot_correlation(
        data,
        'Total Proteome Size',
        category,
        'Total Proteome Size',
        category,
        f'Correlation between Total Proteome Size and {category}'
    )
```

In [62]:

```
## Supplementary Figures S5, S6, S7
```

In [63]:

```
def count_proteins_per_proteome(df, proteome_col):
    return df[proteome_col].value_counts()

# Count proteins in each dataframe
counts_allindomain = count_proteins_per_proteome(df_allindomain, 'Proteome ID')
counts_alloutdomain = count_proteins_per_proteome(df_alloutdomain, 'Proteome ID')
counts_allnodomain = count_proteins_per_proteome(df_allnodomain, 'Proteome ID')
counts_main = count_proteins_per_proteome(main_df, 'Proteome ID')

# Combine all unique Proteome IDs
all_proteomes = (
    counts_allindomain.index
    .union(counts_alloutdomain.index)
    .union(counts_allnodomain.index)
    .union(counts_main.index)
)

# Create a new consolidated DataFrame
consolidated_counts = pd.DataFrame({
    'Proteome ID': all_proteomes
})

consolidated_counts['Complex In Domain'] = consolidated_counts['Proteome ID'].map(counts_allindomain).fillna(0).astype(int)
consolidated_counts['Complex Out Domain'] = consolidated_counts['Proteome ID'].map(counts_alloutdomain).fillna(0).astype(int)
consolidated_counts['Complex No Domain'] = consolidated_counts['Proteome ID'].map(counts_allnodomain).fillna(0).astype(int)
consolidated_counts['Main'] = consolidated_counts['Proteome ID'].map(counts_main).fillna(0).astype(int)

# Display the resulting DataFrame
consolidated_counts.reset_index(drop=True, inplace=True)
```

In [64]:

```
# Merge proteome size information from df_gcatax
data = consolidated_counts.merge(df_gcatax, left_on='Proteome ID', right_on='Proteome ID', how='left')
data.rename(columns={'Phylum': 'Phylum', 'Main': 'Total Proteome Size'}, inplace=True)

# Function to plot correlation for each category
def plot_correlation(data, x, y, xlabel, ylabel, title):
    fig, ax = plt.subplots(figsize=(10, 8))
    
    # Define colors per phylum (ensure phylum_colors is defined somewhere in your code)
    for phylum, color in phylum_colors.items():
        subset = data[data['Phylum'] == phylum]
        ax.scatter(subset[x], subset[y], color=color, label=phylum, alpha=0.6)

    # Label axes and title
    ax.set_xlabel(xlabel)
    ax.set_ylabel(ylabel)
    ax.set_title(title)

    # Calculate Pearson correlation
    r, p = stats.pearsonr(data[x].dropna(), data[y].dropna())
    ax.text(0.05, 0.95, f'r={r:.3f}, p={p:.3g}', ha='left', va='center', transform=ax.transAxes)

    # Add legend
    legend = ax.legend(frameon=False, title='Phylum', bbox_to_anchor=(1.05, 1), loc='upper left')
    plt.tight_layout()

    # Show plot
    plt.box(False)
    plt.show()

# List of categories to plot
categories = [
    'Complex No Domain',
    'Complex Out Domain',
    'Complex In Domain',
]

# Generate plots for each category
for category in categories:
    plot_correlation(
        data,
        'Total Proteome Size',
        category,
        'Total Proteome Size',
        category,
        f'Correlation between Total Proteome Size and {category}'
    )
```
